# Top-Down Ion Mobility Mass Spectrometry Reveals a Disease Associated Conformational Ensemble of Alpha-1-antitrypsin

**DOI:** 10.1021/jacs.4c18139

**Published:** 2025-03-24

**Authors:** Sarah Vickers, Ibrahim Aldobiyan, Sarah M. Lowen, James A. Irving, David A. Lomas, Konstantinos Thalassinos

**Affiliations:** 1 Institute of Structural and Molecular Biology, Division of Biosciences, 4919 University College London, London WC1E 6BT, U.K.; 2 Centre for Respiratory Biology, Division of Medicine, 4919 University College London, London WC1E 6JF, U.K.; 3 Institute of Structural and Molecular Biology Department of Biological Sciences, Birkbeck College London, London WC1E 7JHX, U.K.

## Abstract

Mutants of members of the serpin superfamily can undergo nonamyloid aggregation to form polymeric chains that are associated with disease. This is typified by Z alpha-1-antitrypsin (Glu342Lys) that accumulates as polymers within hepatocytes to cause cirrhosis. We have used ion mobility mass spectrometry and electron-capture dissociation to directly observe and characterize novel intermediates formed during polymerization. Our data are congruent with an ensemble of conformations that are monomeric but maintained in a partially misfolded metastable state in which ∼12% of the molecule at the C-terminus is displaced. The application of these techniques to Z alpha-1-antitrypsin polymers isolated from human liver revealed a molecular species most consistent with a polymer mediated by an intermolecular C-terminal domain insertion. These findings establish a previously unobserved progression of pathogenic structural changes and thereby extend the mechanism of alpha-1-antitrypsin polymerization. They additionally demonstrate the strengths of native top-down ion mobility mass spectrometry in characterizing misfolding intermediates and proteins isolated from human tissue.

## Graphical abstract

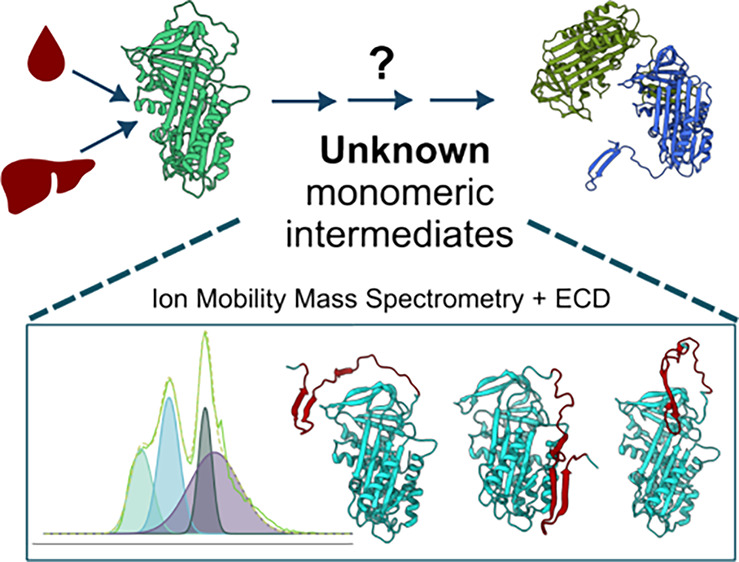

## Introduction

Alpha-1-antitrypsin (AAT) is an archetypal serpin that plays a key role in protecting lung tissue from damage by proteases released during an inflammatory response. It is expressed by hepatocytes in the liver, and normally found at high concentrations circulating in plasma. AAT, in its native metastable state, presents its reactive center loop (RCL) as a substrate for a cognate protease to dock onto and cleave.^1^ Following cleavage, the RCL self-inserts into the central β-sheet A of the molecule, with concomitant translocation of the protease by ∼ 70 Å.^2^ This conformational transition is driven by a substantial gain in thermodynamic stability and in the process, AAT’s inhibitory activity is lost. However, as the mechanism is dependent on a native state with both kinetic stability and thermodynamic instability, the molecule is vulnerable to point mutations that perturb the balance between the two.^3^

Numerous pathogenic mutations of this protein have been described in association with decreased circulating levels, absence of expression or a functional deficiency.^4–6^ Some, notably the Glu342Lys substitution that is encoded by the Z allele, result in the formation of AAT polymers that accumulate in hepatocytes in association with liver disease. Serpin polymers are held together by noncovalent interactions, have a ‘beads-on-a-string’ appearance^7–9^ and display a distinct immunological epitope.^10^ These molecules are functionally inactive. A decrease of AAT below a protective threshold concentration is associated with a predisposition to the development of emphysema.^4^ An elucidation of the polymerization pathway is, therefore, central to an understanding of the pathogenesis of this condition.

A clue as to the earliest, initiating step on the polymerization pathway is found in the X-ray crystal structure of AAT bound to a small molecule inhibitor of polymerization.^11^ Characterization of this compound-bound state by NMR revealed that the structure reflects a minimally perturbed intermediate conformation, termed M*, that is natively populated in the presence of the Z AAT mutation,^12^ whose properties are consistent with observations in spectroscopic^3^ and cysteine scanning experiments.^13,14^ Different models have been advanced for the structural end point of the pathway, with varying degrees of structural and biophysical support, largely based on experiments using recombinantly expressed material.^8,15,16^ Contradictory evidence exists as to the structural progression between these initiating and terminal molecular forms; the intermediate states have been described to adopt an expanded molten globule conformation, to be partially unfolded, folded, to be in a compact non-native and in a near-native state.^16–19^ These incompatible conclusions are largely based on techniques that evaluate the bulk properties of a sample.

While in native mass spectrometry solution phase structures can be maintained, ion mobility mass spectrometry (IMMS) further allows for the separation of species based on their drift time through a region filled with a buffer gas, usually helium or nitrogen.^20–25^ Previous studies have utilized top-down approaches coupled with IMMS to elucidate conformational ensembles of proteins and intermediate states, study aggregation understand protein–protein and protein–ligand interactions, localize binding sites and study proteins from complex tissue samples.^25–35^ We have leveraged the capabilities of cyclic ion mobility mass spectrometry (cIMMS) and electron capture dissociation (ECD) to identify and structurally characterize a misfolding intermediate ensemble of AAT produced during polymerization. This has been compared with a plasma-derived pathogenic variant and has been further extended to assess, for the first time with mass spectrometry, mutant AAT isolated from explanted human liver. Our data are congruent with an ensemble of conformations that are monomeric, have a displaced C-terminus and are therefore consistent with a C-terminal domain swap mechanism.^7,16^ These data further elucidate the structural changes that occur during AAT polymerization.

## Results

### Observation of an Extended Intermediate

Heating of monomeric AAT generates polymers that have been shown to correspond to those present in the liver by negative stain electron microscopy and immunorecognition.^7,10^ Monomeric wild-type AAT (AAT_WT_) and the disease-associated Z variant (AAT_Z_) were isolated from human plasma, and a preliminary analysis using a cyclic ion mobility quadrupole time-of-flight mass spectrometer (cIM-qToF) showed the presence of multiple glycoforms (Supplementary Figure 1) as observed previously.^36–40^

Polymer samples were prepared by heating AAT_WT_ for 48 h at 55 °C (AAT_HEAT_), producing species ranging from monomer to high-order multimers (Supplementary Figure 1C). AAT_WT_ was also incubated at pH 4 and 25 °C for 48 h (AAT_pH_), a condition known to produce polymers immunologically and morphologically distinct from those seen in pathological samples and induced by heat.^19,41^ Masses relating to both monomeric and polymeric assemblies were observed in the native mass spectrometry analysis of AAT_Z_, AAT_HEAT_ and AAT_pH_ (Figure 1), with oligomers up to four subunits in length detected (Figure 1G-I).

**Figure 1:**
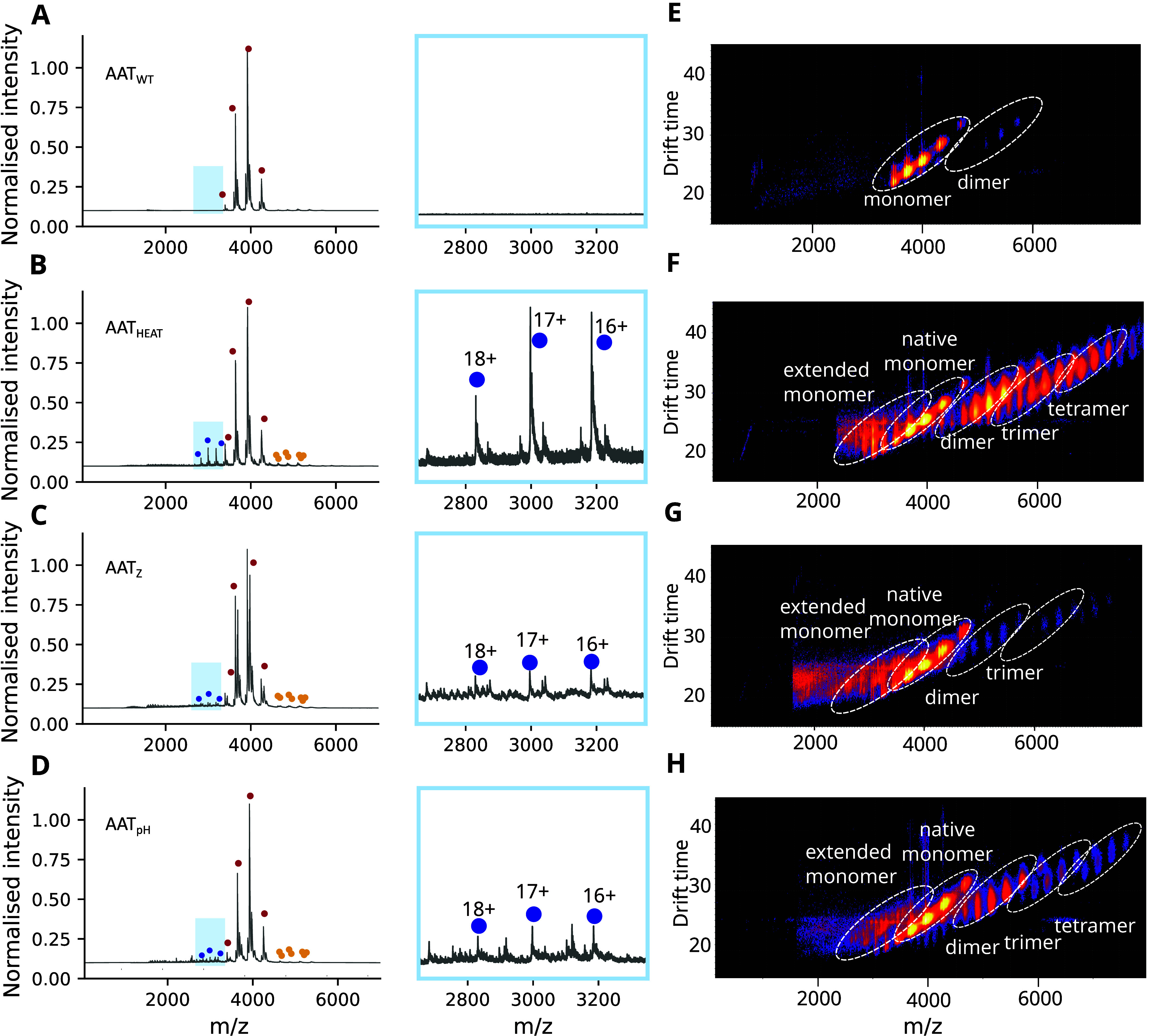
**Mass spectrometry analysis of forms of AAT.** Native mass spectra of (A) AAT_WT_, (B) AAT_HEAT_, (C) AAT_Z_ and (D) AAT_pH_; the region corresponding with higher charge state monomeric species is highlighted in blue and shown in the inset graphs. The red dots correspond to the native conformation of AAT (charge states 12–15+), the yellow dots correspond to the polymer components detected (dimer, trimer and tetramer) while the blue dots indicate the higher charge state species relating to AAT. (E-H) Heat maps showing drift time in the mobility cell against the *m*/*z* values, with detected monomers, low molecular weight polymers and an extended monomeric species highlighted for (E) AAT_WT_, (F) AAT_HEAT_, (G) AAT_Z_ and (H) AAT_pH_. The plots were generated using Driftscope (Waters Corp.) with a logarithmic intensity scale to highlight the presence of low intensity polymers.

In native mass spectrometry experiments, the charge state distribution (CSD) of a protein is influenced by its degree of unfolding.^42,43^ The structure of a protein in solution affects its ionization into the gas phase and therefore the number of protons it retains during the ionization process;^43^ globular proteins have lower charge states, while denatured or disordered proteins have much higher charge states. Proteins that are partially unfolded or have a region of disorder undergo a mechanism of ionization resulting in a charge state distribution intermediate between the two.^42,44^ For AAT_WT_, a single, narrow (12–15+) CSD was observed, consistent with a protein globular in structure (Figure 1A). In contrast, analysis of the AAT_HEAT_ CSD revealed two monomeric charge state distributions, one reporting a native and more compact structure (a CSD of 12–15+) and the other a higher charged and extended species with CSD values from 16 to 18+ (Figure 1B, blue dots). The presence of a higher CSD was also observed in AAT_pH_ (Figure 1D). When comparing the profiles of the low and high CSD within each sample, the same glycosylation pattern and mass was seen (Figure 1, red and blue dots), and thus these differences arose from structural characteristics rather than composition. Therefore, AAT_HEAT_ and AAT_pH_ showed a level of conformational expansion that is not present in the unheated AAT_WT_ sample (Figure 1A).

A comparison with the AAT_Z_ sample also revealed a native AAT_WT_-like low CSD population (red dots), but in common with AAT_HEAT_ and AAT_pH_, it was also found to contain a monomeric, non-native ensemble with a higher CSD (blue dots in Figure 1C) absent from AAT_WT_. Both high and low CSDs within this sample showed the same profile, again consistent with the observed differences arising from structural state rather than component composition. This suggested that the higher CSD reports disease-relevant extended monomeric states of the protein. We therefore sought to characterize these species further.

### The Extended Intermediate Exposes an Epitope Recognized by the 2C1 Monoclonal Antibody

The 2C1 monoclonal antibody (mAb_2C1_) recognizes a cryptic epitope presented by polymers of AAT that form in the liver, as well as on those induced by heat, but does not recognize the functional, native conformation.^10^ This epitope is in a region of the protein associated with conformational change, between helices E and F.^41^ We utilized this specificity to gain structural insights into the extended monomeric ensemble by isolating and analyzing AAT species that were not bound by mAb_2C1_. AAT_HEAT_ was incubated with mAb_2C1_ conjugated to protein G-coated magnetic beads. A native gel (Supplementary Figure 2) showed a shift of the polymeric fraction upon incubation with mAb_2C1_, indicating the polymer components had bound the mAb. Conversely, there was no shift observed of the monomeric subcomponent, and incubation of AAT_HEAT_ with magnetic beads alone showed no change, indicating the shift was due to binding of mAb_2C1_.

Following immunoprecipitation, the supernatant was analyzed using a cIM-qToF. As expected, higher-mass polymer species visible when AAT_HEAT_ was incubated with beads in the absence of mAb_2C1_ (Figure 2A, yellow circles) were no longer visible when incubated in the presence of the antibody (Figure 2B). In contrast, the native-like low CSD monomeric states present in AAT_WT_ (Figure 2C, red circles) and AAT_HEAT_ (Figure 2A, red circles) were not bound and remained detectable (Figure 2B red circles). When considering the higher charge states representative of monomeric extended species, however, the 16+ component decreased in intensity and the 17+ and 18+ components disappeared (Figure 2A-B, blue dots). This indicated that the extended species had been recognized by mAb_2C1_, suggesting that despite being monomeric it contained a structural hallmark of the polymer.

**Figure 2:**
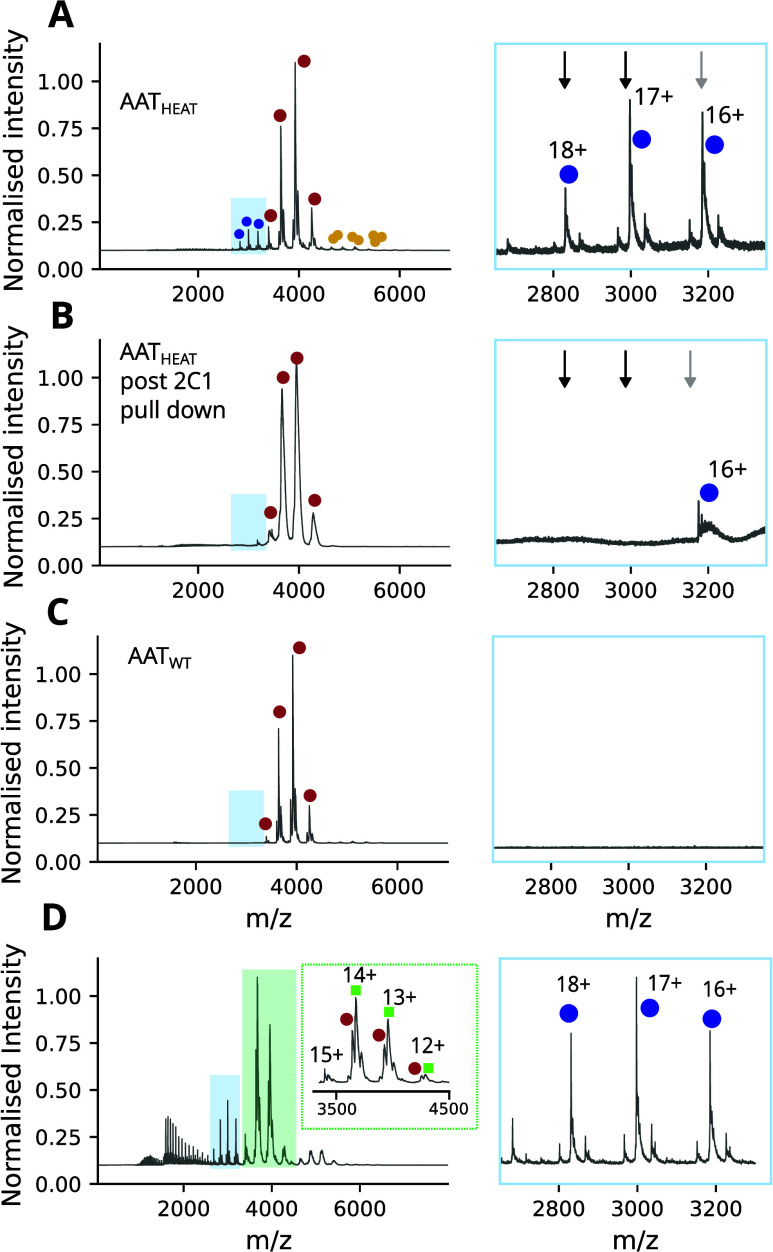
**Investigation of the extended monomeric species through immunorecognition and binding to GSK716.** (A) AAT_HEAT_ samples show the presence of an extended monomeric species. The red dots correspond to the native conformation of AAT (charge states 12–15+), the yellow dots correspond to the polymer components detected (dimer) while the blue dots indicate the higher charge state species relating to AAT. (B) Upon immunoprecipitation with 2C1, the 17+ and 18+ intermediate charge states disappeared (black arrows), the 16+ charge state reduced in intensity (gray arrows), while the ‘native’ charge states 12–15+ were unaffected. (C) In contrast, AAT_WT_ exhibited only 12–15+ charge states. Taken together, these data indicate the binding of the newly identified monomeric intermediate species to the 2C1 antibody showing that this extended state has structured regions and is polymer-like in structure. (D) Investigation of the extended monomeric species binding to the small molecule inhibitor of polymerization. The left panel shows the *m*/*z* spectra of AAT_HEAT_ after incubation with GSK716. The upper inset shows the partial binding of GSK716 to the native charge states (12–15+ of AAT); red circles denote native AAT and green squares show native AAT bound to GSK716. The side panel shows the extended charge states (blue dots) with no *m*/*z* shift to indicate the binding of GSK716.

### The Extended Intermediate Does Not Correspond to the M* Intermediate

AAT_Z_ natively populates an alternate conformation, M*, representing the precursor that initiates polymer formation. A small molecule inhibitor of polymerization, GSK716, has been shown to bind to a cryptic site within the breach region,^11^ stabilizing this early intermediate.^12^ The activity of the small molecule both in vitro and in vivo emphasizes the role that the M* conformation plays in polymerization both during folding and upon destabilization of the native molecule. GSK716 can therefore act as a sensitive reporter for the presence of M*. Incubation of AAT_HEAT_ with GSK716 showed a mass increase due to ligand binding only for the native-like monomeric subcomponents with a lower 12–14+ charge state (Figure 2D, green squares); no difference was detected for the monomeric extended 16–18+ higher charge states (Figure 2D, blue dots).

It has been observed that GSK716 does not bind to subunits within the polymer.^11^ Taken together, these data indicate that we have identified a monomeric ensemble with an extended conformation that is natively formed by the Z variant and adopted by wild-type AAT during heat-induced polymerization. This species shares a cryptic epitope with the AAT polymer but does not correspond to the near-native M* intermediate.

### Multiple Conformational States Are Present in AAT_HEAT_ and AAT_Z_ Samples

To obtain structural information on the conformational ensemble induced by heating and natively present in the Z variant, we performed CCS characterisations of each of the charge states of AAT_HEAT_ and AAT_Z_ with reference to AAT_WT_ (Figure 3). Analysis was performed using the cIM-qToF to obtain arrival time distributions for each charge state, which were then converted to CCS by calibration using four protein standards (see methods) and deconvoluted by fitting Gaussian functions. The lower charge states displayed narrower, predominantly single conformations indicating a degree of conformational stability with a CCS common to all samples (Figure 3, 12–14+ charge states, labeled α). For AAT_HEAT_, four distinct non-native conformations were identified (Figure 3, AAT_HEAT_), with the higher charge states displaying multiple, progressively extended forms (Figure 3, 15–18+, β, δ, and γ). The AAT_Z_ protein also showed four states, although the relative contributions to the profile varied by charge state from AAT_HEAT_ (Figure 3, AAT_Z_).

**Figure 3:**
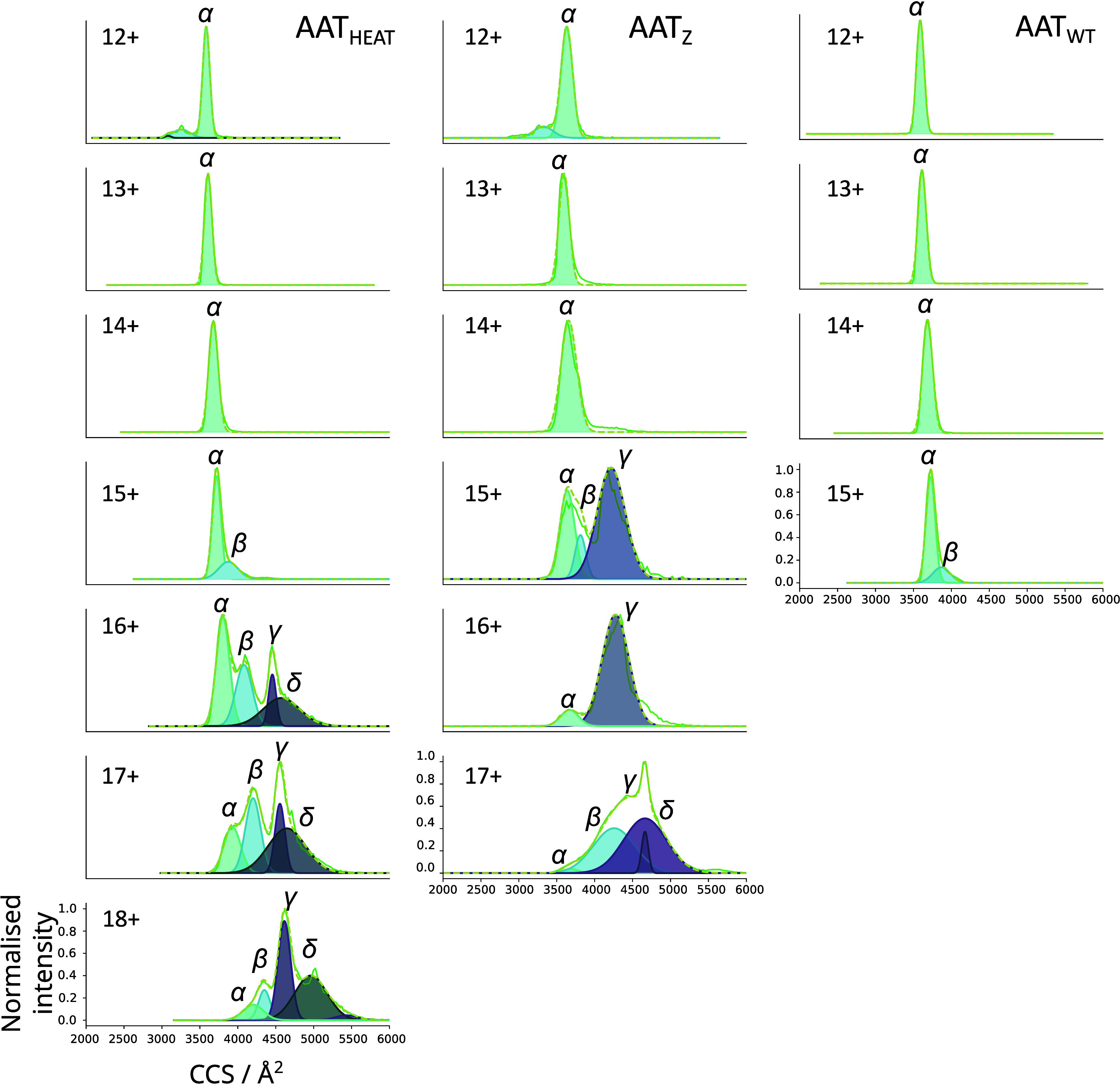
**Collision cross sections of native and non-native monomer charge states of AAT**_**WT**_ **, AAT**_**HEAT**_ **and AAT**_**Z**_. Drift times of each species were converted to CCS using calibration with IMSCal19 ^45^ and peaks fitted using the Gaussian function in Origin Pro (2021). The 12–14+ charge states of all samples show a similar compact CCS (α), while 15–18+ of the AAT_HEAT_ and 15–17+ AAT_Z_ show a larger conformational ensemble (β,γ, and δ) The CCS profiles of the higher charge states could be fit by multiple Gaussians, reflecting their conformational heterogeneity and flexibility.

### The Extended Ensemble Has an Exposed C-terminus and a RCL That Is Not Fully Incorporated

Different mass spectrometry fragmentation methods can provide a rich description of the structural state of individual components, even within a complex mixture. In electron capture dissociation (ECD), fragmentation of the peptide backbone occurs when the protonated analyte captures an electron, causing the formation of c and z fragment ions.^46–50^ Crucially, ECD can reveal information on the surface accessibility of different regions of a protein; during this process the conformation of the molecule is not impacted, with the resultant fragmentation of the peptide backbone occurring only after the electron capture step.^47^ Within our ECD experiments, a single charge state was first isolated in the quadrupole, and then separated according to its structure in the mobility cell, before entering the ECD cell for fragmentation. In these experiments, the AAT_WT_ species with a 15+ charge state was used as a native-like reference, while the conformationally heterogeneous 17+ charge state of AAT_HEAT_ was used to study the extended monomeric state. The 15+ state showed minimal fragmentation at both the C- and N-termini (Figure 4A, lower). In contrast, for the 17+ extended intermediate, extensive fragmentation was observed from residue A349 to the C-terminus, indicating these residues were readily accessible and not fully folded (Figure 4A, upper).

**Figure 4:**
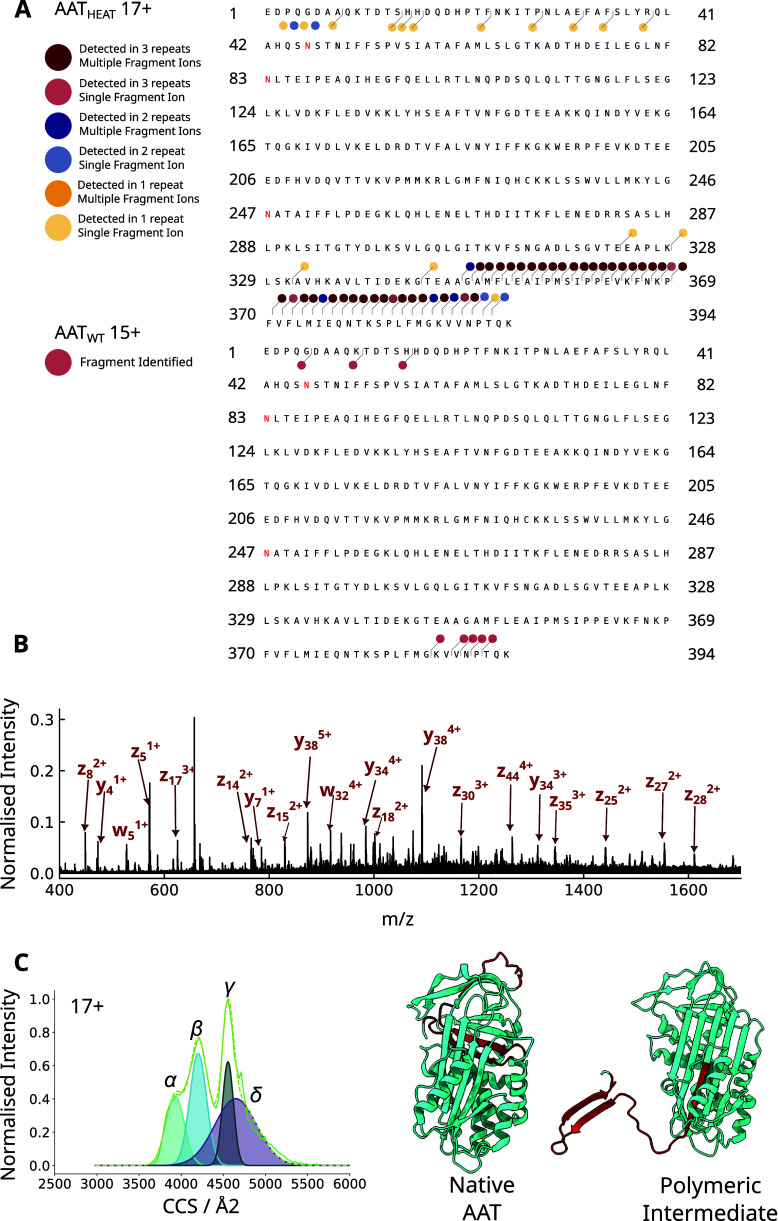
**Electron capture dissociation (ECD) to investigate the extended monomeric species. (**A) ECD of AAT_HEAT_ (17+) (above) and AAT_WT_ (15+) (below). The ECD fragments are labeled on the amino acid sequence of AAT The colored dots denote the fragment ions (charge states and b/y/c/z) detected. Dark red dots indicate multiple fragment ions were detected in all 3 repeats, and light red, blue and yellow indicate that a single fragment ion was detected for 3 repeats, 2 repeats and 1 repeat, respectively. (B) Mass spectrum of AAT_HEAT_ (17+) after ECD with the most intense fragments labeled. (C) *Left*, the calculated CCS distributions of the 17+ charge state of AAT_HEAT_ is shown. *Right*, the identified ECD fragments for the 17+ species are shown in red on the crystal structures of wildtype AAT (native -PDB: 1QLP)^16,53^ and a subunit of an artificial AAT polymer (intermediate - PDB: 3T1P).^16^

To preclude the possibility of charge effects in the ECD process on the observed fragmentation of the 17+ state of AAT_HEAT_, the 15+ state, which contained both native-like and extended components, was also assessed (Supplementary Figure 3). While this charge state had a low intensity relative to 17+, reducing the number of detectable fragments, fragmentation was again seen in the extended ensemble at P357 but not in the native-like subcomponent, giving further support for presence of an exposed C-terminus in the extended monomeric state.^51,52^

### Probing the Structure of the Extended Monomeric State

To investigate the implications of these ECD fragment data for the extended ensemble, we mapped the observed fragments onto the crystal structures of native AAT monomer and a subunit of the C-terminal polymer crystal structure (Figure 4C, right).^16,53^ The native structure was found to be incompatible with the fragments observed, as in this state the C-terminal domain would be incorporated within the molecule, and thus unavailable for fragmentation. While the RCL is solvent-exposed in the native conformation, the resulting ∼ 4 kDa fragment would not be observed without denaturation of the protein (Figure 4C, native AAT).

It has been hypothesized that a C-terminus-displaced, fully-RCL inserted conformation is a potential intermediate on the polymerization pathway.^16^ This would be expected to show protection against fragmentation before residue ∼ 359. Interestingly, the fragmentation observed for the 17+ extended monomeric species reached a point 10 residues N-terminal to this, in the middle of strand 5A (Figure 4C, right panel). The fragmentation pattern would indicate that the RCL incorporation is incomplete relative to that seen in the C-terminal polymer structure.

### Polymers Isolated from Human Liver Tissue Contain a Monomeric Species with a Displaced C-terminus

We next wanted to determine whether we could identify this unfolded species in *ex vivo* liver tissue samples from patients homozygous for the Z mutation, the most clinically relevant phenotype. AAT purified from the *ex vivo* liver tissue of a Z AAT homozygote (AAT_LIVER_) was analyzed with native IMMS (Figure 5). From the resulting mobiligrams, oligomers as large as tetramers could be resolved (Figure 5B). However, due to their inherent heterogeneity, the extensive overlap of species, presence of degradation products and low ion count, these samples were challenging to analyze.

**Figure 5:**
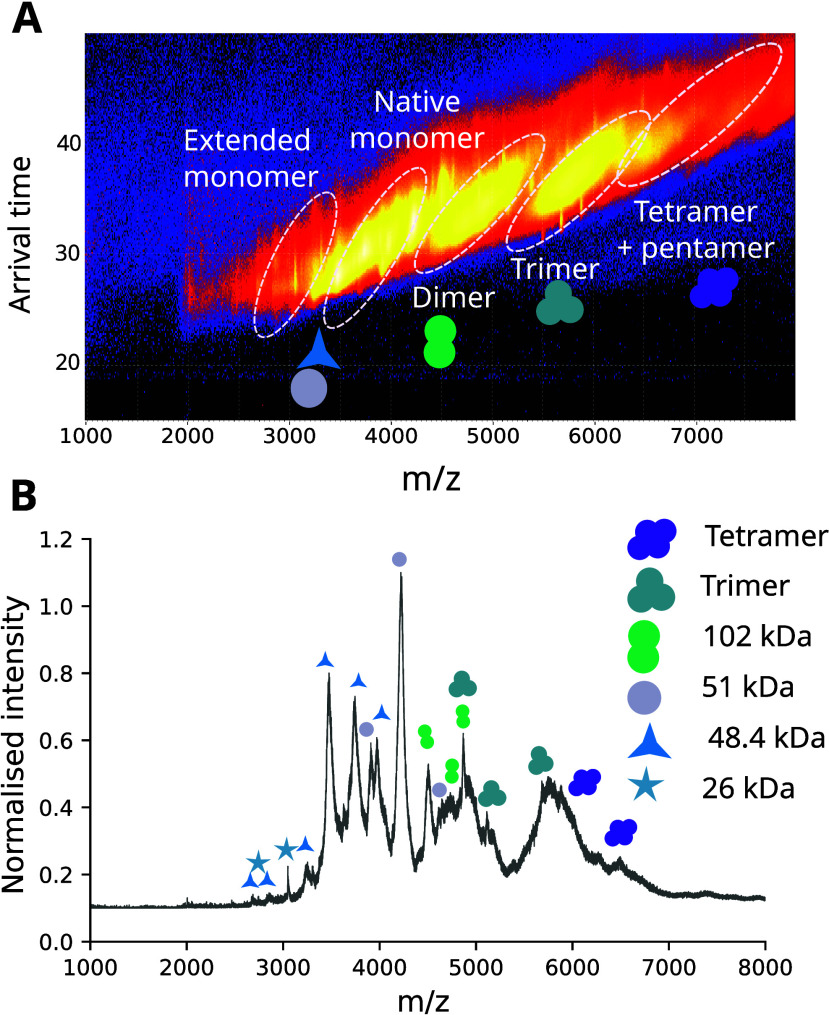
**Deconvolution of mass spectra of** *ex vivo* **AAT from the liver tissue of a Z AAT homozygote.** (A) Heat map of data acquired on a cIM-qToF instrument showing mobility versus *m*/*z*, with monomeric to pentameric species labeled; data were plotted in Driftscope, with intensity on a log scale. (B) Mass spectra of *ex vivo* liver samples acquired on a cIM-qToF, with identified masses labeled.

In addition to fragmentation, the capture of electrons in an ECD cell can be used to charge-reduce an isolated charge state of a protein and thereby help deconvolute complex heterogeneous mass spectra.^54,55^ Using this approach to charge-reduce quadrupole-isolated species (Supplementary Figure 4), we were able to confidently identify a dimeric subunit of AAT liver polymer (102 kDa) as well as two AAT monomeric species (48 and 51 kDa). The lower-mass species was confirmed to not be truncated, as fragments at both the C- and N- terminus were identified. The polymers present in liver tissue are known not to have fully transited the cellular secretory pathway and therefore do not have a mature glycosylation profile, which might explain the presence of the lower mass species. The resolved monomeric component exhibited charge states up to 18+. There were also multiple lower-mass AAT species, which are most likely degradation products.

To understand the structure of the monomeric species of Z AAT from *ex vivo* liver, the 14+ charge state of the species relating to 48 kDa AAT was isolated and subjected to ECD (Figure 6). The arrival time distribution of this species showed some conformational heterogeneity with at least two conformations present in the ensemble, although this was lower than the ensemble observed in AAT_HEAT_ and AAT_Z_. There was extensive fragmentation in the C-terminal domain observed up to Ala 355, which is partially buried in the fully-RCL-inserted state. Therefore, strikingly, the ECD fragment map of this tissue-derived species strongly resembled that of the 17+ extended monomeric species in AAT_HEAT_, albeit with fragments not extending to as much of the inserted strand 5A.

**Figure 6:**
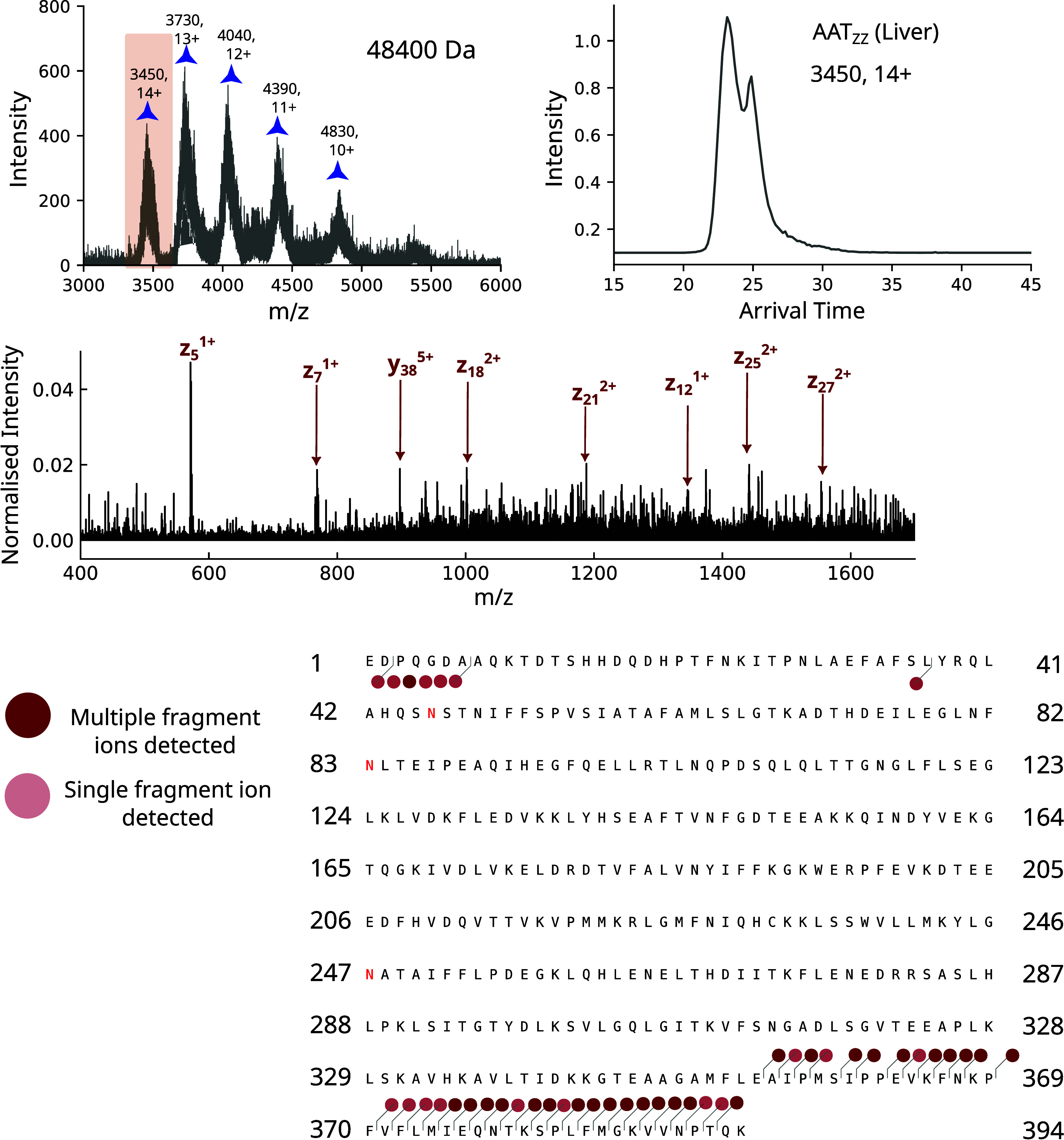
**Investigation of AAT monomer isolated from explanted liver tissue from a Z AAT homozygote.** A species relating to AAT with a mass of 48400 Da was identified using electron capture charge reduction experiments. The 14+ charge state of this species was isolated, as this was more abundant and with less overlap than the corresponding 17+ charge state, and was mobility analyzed (upper right panel). The mass spectrum of the species after ECD (middle panel) had the most intense assigned fragments labeled. Fragments with multiple ions identified are shown in dark red on the AAT amino acid sequence, while fragments with one ion identified are shown in pale red. Fragment identification was performed with ExDViewer.^56^

One advantage of the ECD approach is that the process of electron capture – revealing accessible regions of the structure – and dissociation occur in distinct phases, minimizing disruption of the structure. Nevertheless, AAT polymers have been robustly demonstrated in the literature to exhibit extreme stability to destabilizing conditions.^57^ We made use of this property to establish whether it would be possible to observe discrete fragmentation within the dimer component of AAT_HEAT_, AAT_pH_ and AAT_LIVER_ at increasing levels of collision energy. In AAT_HEAT_ and AAT_LIVER_, a fragment at 1379 *m*/*z* was observed, corresponding with a y36 3+ C-terminal fragment, when an energy was applied at which the dimers of all three samples remained intact without dissociation to their constituent monomers (Figure 7). The vulnerability of this C-terminal region to fragmentation without dissociation to the monomer, and the lack of formation in AAT_pH_ which is known to be structurally distinct,^19^ suggests that it was exposed and reflective of the pathological polymer. It is noteworthy that an exposed C-terminus is inconsistent with a circular, self-terminating polymer configuration as has previously been proposed.^16^

**Figure 7:**
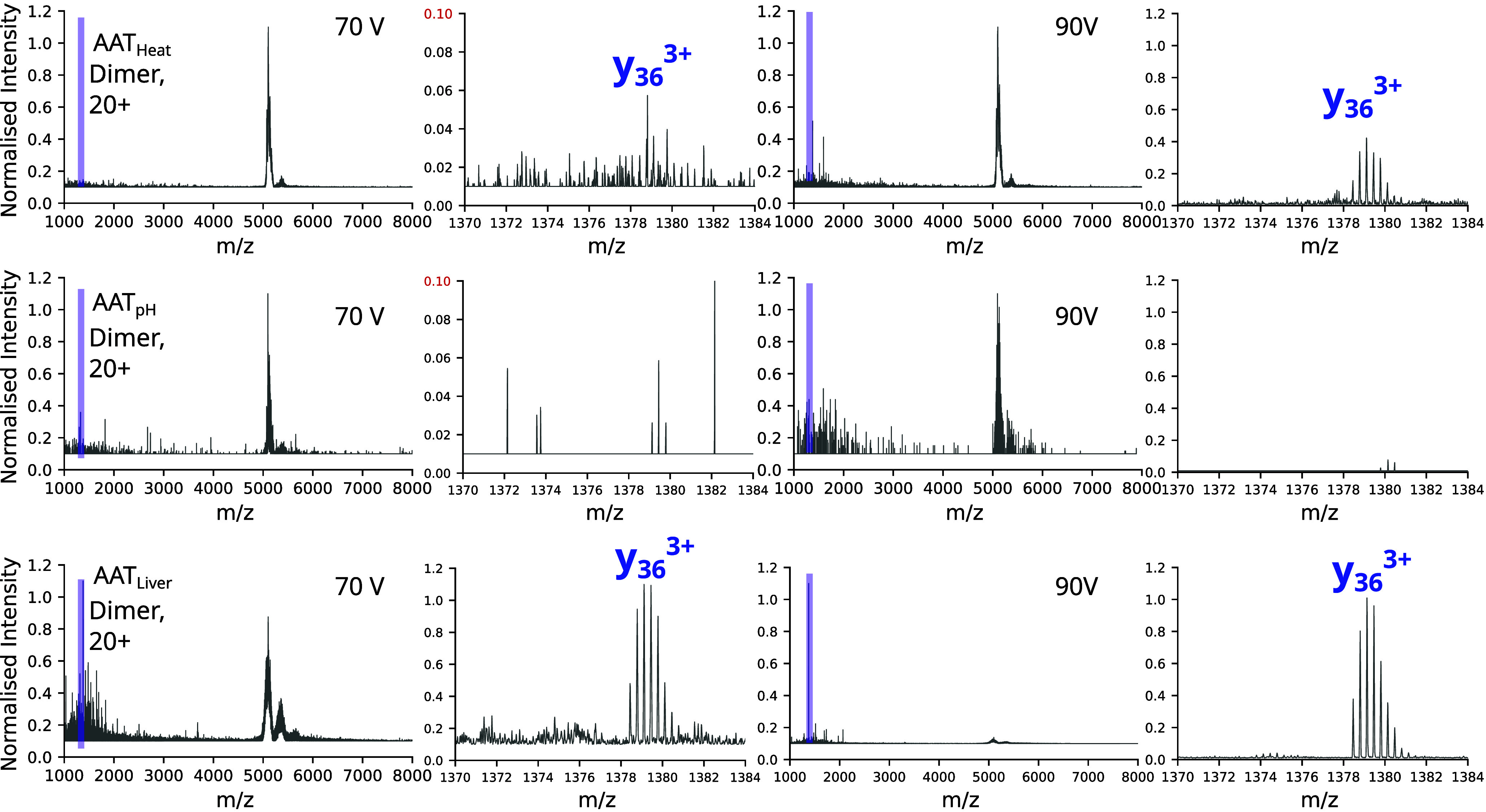
**Collisional activation of AAT**_**HEAT**_ **, AAT**_**pH**_ **and AAT**_**LIVER**_ **dimers.** Samples were subjected to 70 and 90 V of CA energy in the trap, respectively. Insets show the presence (or absence) of the *m*/*z* 1379 y36 3+ fragment at the C-terminus of AAT.

## Discussion

To understand the mechanistic basis for pathologies associated with protein accumulation, it is necessary to investigate the structural intermediates that are navigated in the process. These intermediates can be viable targets for therapeutic intervention^12^ or the development of clinical diagnostics.^58–60^ Due to their transient nature, however, these states are difficult to isolate and characterize. AAT traverses multiple intermediates during polymerization, but little is known about their structure.^7^ We have used cyclic ion mobility mass spectrometry to isolate and characterize an extended intermediate that that is formed during polymerization of AAT. This ensemble was found to be structured, and to exhibit a hallmark of the polymer form, due to its binding by the mAb_2C1_ monoclonal antibody that recognizes a cryptic epitope present on liver-derived polymers. The lack of recognition by a small molecule that selectively targets an early intermediate, referred to as M*, in addition to the monomeric nature of its components, suggest that this ensemble reflects intermediates that arise later on the polymerization pathway.

Traditional characterization of complex species by MS has relied on a bottom-up approach dependent on sample treatment prior to analysis, for example by chemical derivatization or enzymatic modification. Top-down MS in contrast performs fragmentation of the intact protein within the instrument. It showed the monomeric intermediate population to be well-structured and provided evidence most consistent with a polymerization mechanism in which the C-terminus is displaced from its canonical position in a donor molecule and is primed to incorporate into the equivalent position in an adjacent molecule. The susceptibility of almost all sites within the C-terminus to fragmentation demonstrates that it is displaced from its protected cognate position within the molecule.

The data further reconcile the paradox between a polymerization mechanism predicated on self-insertion of the RCL, and the observation that during folding AAT does not self-incorporate this region, despite the fact that it is the last part of the protein to emerge from the ribosome/translocon complex during synthesis.^61^ A portion of this loop that would be protected from ECD if it was in a fully inserted conformation was found instead to be susceptible to fragmentation, revealing the presence of a population in which the loop has not been able to fully incorporate into β-sheet A (Figure 4).

A monomeric component of material extracted from liver tissue was found to have a displaced C-terminus (Figure 6). The arrival time distribution of this species had less conformational heterogeneity and more compaction than the extended intermediate seen in AAT_HEAT,_ while having more extension than the low charge state monomers in AAT_WT_. Coupled with the observation of C-terminal fragments no further than seen with full insertion, the data indicate that the monomeric species in AAT_LIVER_ most likely represents a subunit from liver polymer. This could be produced either by degradative processes within the cell or during purification. The conclusion that the extended monomer in AAT_HEAT_ is a dynamic intermediate with a partially incorporated RCL, and the monomer in AAT is likely a subunit from polymers with a fully inserted RCL, is collectively consistent with the C-terminal mechanism of polymerization.

The observation of fragmentation in discrete regions of the molecule, coupled with the recognition by a conformationally selective antibody, mAb_2C1_, strongly suggest that the identified intermediate ensemble is well-structured. The absence of the species in AAT_WT_ shows that this extended charge state is not a result of the electrospray ionization process. Thus, we propose that ionization occurs via an intermediate mechanism in which the C-terminal region ionises by the chain ejection model and the rest of the protein ionises via the charged residue model as proposed by Beveridge et al.^42–44,62^

Nevertheless, the higher mass-to-charge states support more extended conformations of the molecule than observed with the native state. The cryptic epitope recognized by mAb_2C1_ is situated in the region between the E and F helices,^41^ which undergoes substantial changes during expansion of β-sheet A. The recognition of this species by this antibody, the lack of interaction with GSK716, which binds to an early stage polymerization intermediate but not to polymers,^11,12^ and the fragmentation profile collectively indicate that the intermediate described here is structurally distinct from the globular and compact M*.^11,63^ The data suggest that this extended intermediate arises further along the polymerization pathway,^7^ has an expanded β-sheet A, a polymer-like structure in the breach region, but only partial incorporation of the RCL. The native presence of this conformational ensemble in monomeric AAT isolated from the plasma of individuals with the ZZ genotype, but not unheated wild-type plasma, indicates that they are pathologically relevant.

Our data are consistent with a polymerization pathway mediated by a C-terminal domain swap as shown in Figure 8. The direct evidence for a novel intermediate ensemble obtained in this study extends the mechanism of polymerization, whereby the structural progression proceeds first through a compact intermediate (which binds GSK716, M*) before undergoing a change yielding an ensemble of intermediates with a released RCL and C-terminus which do not bind GSK716. An acceptor AAT molecule then incorporates a donor C-terminus before self-insertion of its RCL can occur. Crucially, all the evidence that we present is using *ex vivo* systems, while previous intermediates have been extrapolated from studies using *in vitro* systems.

**Figure 8:**
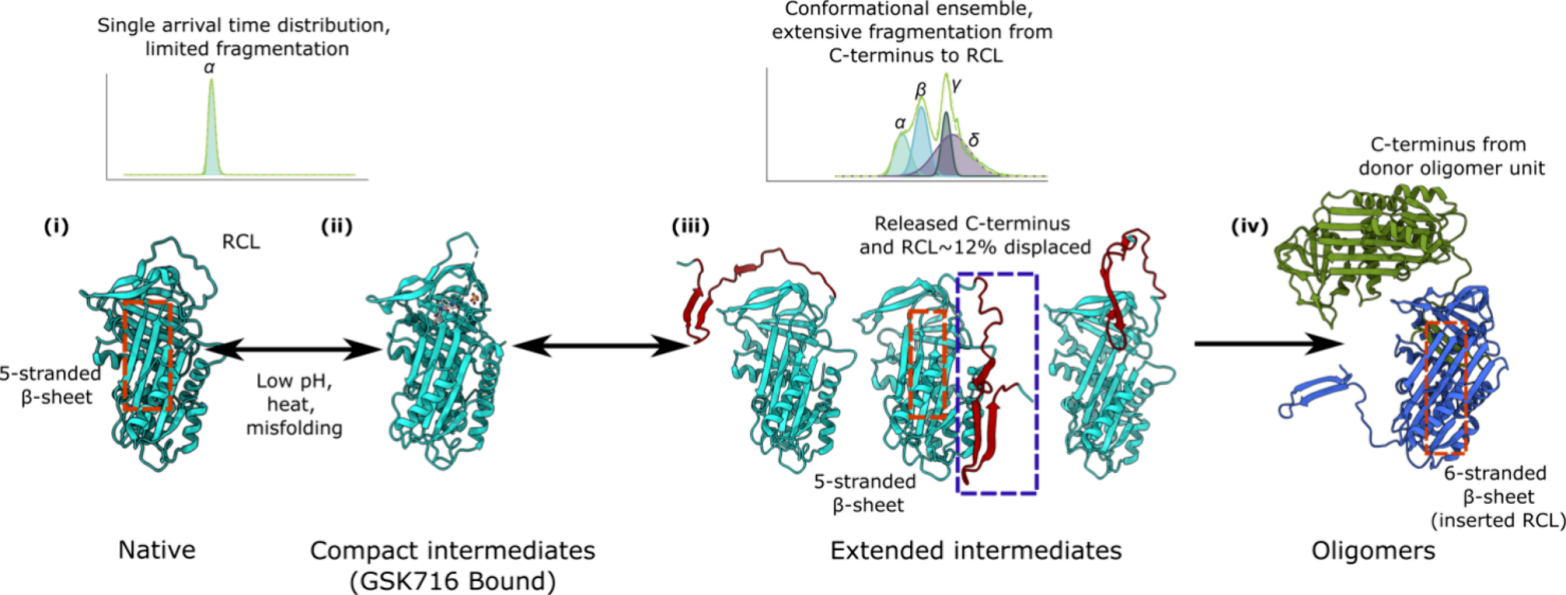
**The characteristics of the intermediate ensemble are consistent with a C-terminal mechanism of polymerization.** (i) Native folded AAT (PBD: 1QLP^53^) has a 5-stranded β-sheet A with an accessible RCL. Conformational analysis of this species shows a narrow, singular population. AAT polymers can form through incubation at high temperature, low pH or in the presence of a point mutation such as that of the pathological Z variant. A previously defined compact intermediate (ii) has been characterized by binding to the small molecule GSK716 (PDB: 7AEL),^11,12,63^ which we propose is earlier in the polymerization pathway. We have identified an extended intermediate species (iii) that is conformationally heterogeneous and propose that these intermediate conformations have a displaced C-terminus and a RCL that is not in the fully inserted conformation. Following adoption of this intermediate, polymerization proceeds with an acceptor AAT molecule incorporating the C-terminus of another molecule, releasing the kinetic lock and allowing self-insertion of its RCL.

## Conclusion

Our approach illustrates the novel insights that the unique combination of cyclic ion mobility and fragmentation can provide in the characterization of low abundance, disease-relevant molecular conformations, including species obtained from *ex vivo* samples. This strategy could in the future be applied to the study of other proteins that misfold and aggregate but which are difficult to study by other biophysical and structural approaches.

## Materials and Methods

### Preparation of Samples from Plasma (AAT_WT_ and AAT_Z_)

The purification of AAT has been described in detail previously.^64^ In brief, filtered plasma from human donors was applied to an Alpha-1-Antitrypsin Select column (Cytiva) equilibrated in PBS, washed to baseline in PBS, and eluted using 2 M MgCl_2_, 50 mM Tris, pH 7.4. Combined fractions were dialyzed overnight against 20 mM Tris pH 8.0, 100 mM β-mercaptoethanol was added, and the sample applied to a 5 mL HiTrap Q column (Cytiva). A 0–0.5 M sodium chloride gradient in 20 mM Tris pH 8.0, 0.02% w/v sodium azide buffer was applied across 80 mL, with monomeric AAT eluting at around 150 mM NaCl. The fractions were pooled and buffer exchanged into PBS, diluted to 1 mg/mL and stored at −80 °C. All chromatography steps were performed using an AKTA Pure instrument (Cytiva) and sample purity was assessed by SDS-PAGE and nondenaturing PAGE (Life Technologies). For the experimental work shown, plasma was from the same purification (and therefore donor).

To produce polymers, AAT_WT_ at 0.4 mg/mL was incubated in PBS for 48 h at 55 °C in a water bath (AAT_HEAT_) or at pH 4 in PBS for 48 h at 25 °C (AAT_pH_). All samples were buffer exchanged into 100 mM ammonium acetate pH 7.4 using Amicon 30 kDa MWCO centrifugal filters (Merck) at 4 °C. Samples were aliquoted and stored at −80 °C, at 0.5 mg/mL for AAT monomers or 1.5 mg/mL for polymers.

### Preparation of Samples from *Ex Vivo* Liver (AAT_ZZ liver_)

Sections (∼150g) of explanted liver tissue from a ZZ AAT homozygote who had undergone transplantation for Z AAT deficiency-associated cirrhosis were thinly sliced and ground on ice using a 50 mL CS1 Tissue Grinder (DWK Life Sciences), and incubated at 37 °C at 200 rpm for 1 h in 30 mL of Hanks balanced salt solution (140 mM NaCl, 5 mM KCl, 1 mM CaCl_2_, 0.4 mM MgSO_4_, 0.3 mM Na_2_HPO_4_, 6 mM Glucose, 4 mM NaHCO_3_) with 15 mg collagenase (Type 1A, Merck). The digested sample was filtered through nylon mesh and the filtrate centrifuged at 4 °C for 30 min at 3000g. The pellet was resuspended in 5 mL of PBS, sonicated (Q500 Sonicator, QSonica) in a chilled water bath at 50% amplitude for 60 s, applied on top of 500 mL 1.3 M sucrose at 4 °C and centrifuged at 16000g at 4 °C for 1 h. The supernatant was discarded and the pellet was repeatedly washed in PBS by resuspension and centrifugation for 15 min at 16000g and 4 °C until the supernatant appeared clear.

Soluble polymers were released from the resulting inclusion bodies by sonication in a chilled water bath at 50% amplitude for 15 s on/30 s off for 10 min, with nonextractable components removed by repeated resuspension and centrifugation at 16000g at 4 °C for 15 min. The sample was applied to a 5 mL HiTrap Q column (Cytiva) and purified by the same protocol as given for the preparation of samples from plasma above.

### Antibody Pull-Down Experiment

A 25 μL suspension of magnetic Protein G-coated Dynabeads (ThermoFisher Scientific) was washed five times with 100 mM ammonium acetate. Samples (5 μL of 1 mg/mL) and mAb_2C1_ (15 μL of 1 mg/mL) were mixed with the magnetic beads in a volume of 25 μL PBS incubated using a Stuart Rotator SB3 benchtop rotating mixer at 50 rpm for 30 min at room temperature after which the supernatant was retained for MS analysis.

### Mass Spectrometry

Samples were analyzed using a SELECT SERIES Cyclic ion mobility q-TOF (cIM-qToF) (Waters Corp.) fitted with an ECD cell. Samples were infused into the mass spectrometer via nanoelectrospray ionization (nESI) using an in-house pulled borosilicate glass capillary coated in gold (Sutter P-97 flaming brown pipet puller). Typical MS parameters were: capillary = 0.9–1.5 kV, cone = 50 V, source offset = 30 V, source temperature = 40 °C. trap = 6 V, transfer = 4 V, quad profile = 2000/4000/6000.

For standard ion mobility experiments, charge states were isolated in the quadrupole before entering the mobility cell. Standard mobility parameters were: traveling wave static height = 30 V, traveling wave velocity = 375 m/s, separate wave height = 5, pushes per pin = 1, bins = 200, inject time = 10 ms, separate time = 5 ms, eject and acquire = 45 ms. These settings were kept consistent for all samples and CCS calibrants (Conconavalin A, beta lactoglobulin and bovine serum albumin). For activation, the trap voltage was increased in 2 or 10 V increments to study the change in arrival time as supplemental energy increased.

For ECD, the ECD filament was switched to ‘on’ (current = 2.4 A) and the cell settings set to L1 = 2, L2 = −15; LM3 = 9.5; L4 = 9.3; FB = 4.2; LM5 = 47.5; L6 = −19.0; L7 = 2.0. The transfer was increased to 34 V to release fragments. A control experiment with the transfer at 34 V showed that no fragments were released, showing these fragments are related to the ECD cell. Data were acquired for 30 min due to the low ion count when the ECD cell is on.

For collision activation experiments on the dimer, the 20+ charge state (5100 *m*/*z*) was isolated in the quadrupole before a supplemental activation energy (70 or 90 V of supplemental energy) was applied premobility in the trap.

## Supplementary Material



## Notes

Additional experimental details for CCS calibration and data processing (Section 1); deconvoluted mass spectra of AAT WT and Z (Figure 1); native gel showing the binding of 2C1 and beads (Figure 2); slicing and ECD of 15+ charge state of AAT heat in comparison to AAT WT (Figure 3); mass spectra from ECD charge reduction experiments (Figure 4) (PDF)

## Abbreviations

AAT: Alpha 1 antitrypsin
CCS: Collision cross section
cIMMS: Cyclic ion mobility mass spectrometry
ECD: Electron capture dissociation
RCL: Reactive center loop
