# Supplementary material for: Top-Down Ion Mobility Mass Spectrometry Reveals a Disease Associated Conformational Ensemble of Alpha-1-antitrypsin
Source: J Am Chem Soc. 2025 Mar 24;147(20):16909–21. doi: 10.1021/jacs.4c18139 (PMC12100655; doi:10.1021/jacs.4c18139)
Supplement: Supplementary file 1 [file ja4c18139_si_001.pdf]

Top-down ion mobility mass spectrometry reveals a disease associated conformational ensemble of alpha-1-antitrypsin

Sarah Vickers[1,2,3], Ibrahim Aldobiyan[2,3,], Sarah M. Lowen[2,3], James A. Irving[2,3]*, David A. Lomas[,2,3]*, Konstantinos Thalassinos[1,3]*

[1] Institute of Structural and Molecular Biology, Division of Biosciences, University College London, WC1E 7JE, London, U.K.

[2] Centre for Respiratory Biology, Division of Medicine, University College London, WC1E 6JF, London, U.K.

[3] Institute of Structural and Molecular Biology Department of Biological Sciences, Birkbeck College London, WC1E 7JE London, U.K.

[4] Department of Biochemistry, College of Science, King Saud University, PO Box 2455, Riyadh 11451, Saudi Arabia.

Description of Supplementary Materials

1. **Additional Experimental details for CCS calibration and data processing**
2. **Supplementary figure showing deconvoluted mass spectra of AAT WT and Z.**
3. **Supplementary figure of native gel showing the binding of 2C1 and beads.**
4. **Supplementary figure showing slicing and ECD of 15+ charge state of AAT heat in comparison to AAT WT.**
5. **Supplementary figure showing mass spectra from ECD charge reduction experiments.**

Supplementary Materials

Supplementary Methods

CCS Calibration

CCS calibration was performed using the following protein standards, acquired from Sigma Aldrich and prepared for MS using the method described above: β-lactoglobulin, cytochrome C, concanavalin A, avidin and bovine serum albumin. Calibrants were made up to 40 μM in buffer S and buffer exchanged three times using Amicon 30kDa MWCO filters (Merck). Charge states of samples were isolated, and the mobility acquired. Sample arrival times and the corresponding literature CCS in Nitrogen[78,79] were inputted to IMSCal software (Waters Corp.)[63] for calibration. Arrival time data of samples were inputted to the software to convert to CCS.

Data Processing

Data were viewed on Masslynx (Waters Corp.) and if necessary were smoothed using the Savitzky-Golay smoothing method with a window size ≥2 (up to 10 for complex samples) and a smooth number of 1. For the deconvolution of mass spectra, either charge states were used to convert from m/z to mass manually or the MaxEnt 1 module of masslynx was used [80]. For the deconvolution of the glycoforms of monomeric AAT, the mass range was set to 49-55 kDa, a resolution of 1.5 Da/channel and iteration was set to convergence.

For CCS comparisons, gaussian peaks were fitted using the multiple peak fitter of Origin Pro (2021) [81], iterating peaks to convergence and within a r^2 value of >0.99 for AAT_{WT} and AAT_{HEAT} and >0.969 for AAT_{Z}.

For deconvolution and assignment of AAT_{HEAT} and AAT_{WT} ECD fragments, ExDViewer (Agilent) was used[82]. Spectra were averaged and an MS2 m/z tolerance of 20 ppm was used and restrictive ion identification quality settings were applied. The glycosylated native sequence of AAT_{WT} was used for matching, with the settings: peak picking signal to noise: 0.1; match tolerance: 20.0 ppm; minimum score: 0.7; ion types: a, b, c, d,w,y,z. Both c/z and b/y fragments were observed in ECD data which has been reported previously [64,67]. Fragments in figures were assigned based on ions being observed as either both c/z and b/y ions or in multiple charge states (to meet 'multiple fragment ions' threshold). For the *ex-vivo* liver species, the fragmentation spectrum was deconvoluted using ExDViewer and fragments were assigned manually based on predicted fragments from the AAT sequence using ms-product [83]. Tables of fragment assignments are shown below.

Supplementary Figures

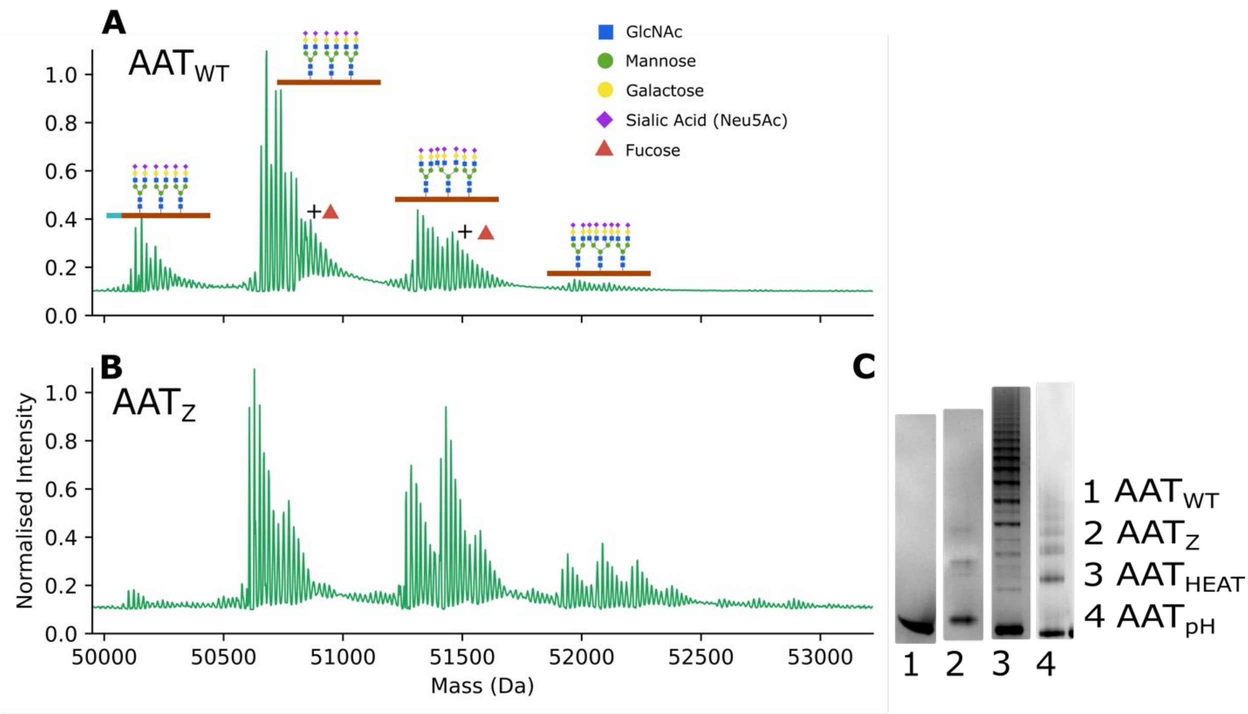

Supplementary Figure 1: Deconvoluted Mass Spectra of AAT_{WT} (A) and AAT_{Z} (B). Three major glycoforms were identified alongside a glycoform with a truncated N terminus. Fucosylated glycoforms were also observed. C) The purity of the samples (AAT_{WT}, AAT_{Z}, AAT_{pH} and AAT_{HEAT}) and the presence of oligomers in solution was confirmed on a 3-12% w-v acrylamide bis-Tris non-denaturing PAGE. 2 ug of protein were loaded in each lane.

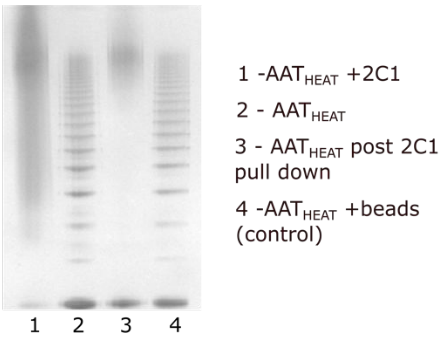

Supplementary Figure 2: 3-12% (w/v) acrylamide bis-Tris non-denaturing PAGE showing: AAT_{HEAT} incubated with mAb_{2C1}, AAT_{HEAT} alone, AAT_{HEAT} following immunoprecipitation with mAb_{2C1} and magnetic beads and AAT_{HEAT} incubated with beads alone as a control, in lanes 1-4, respectively.

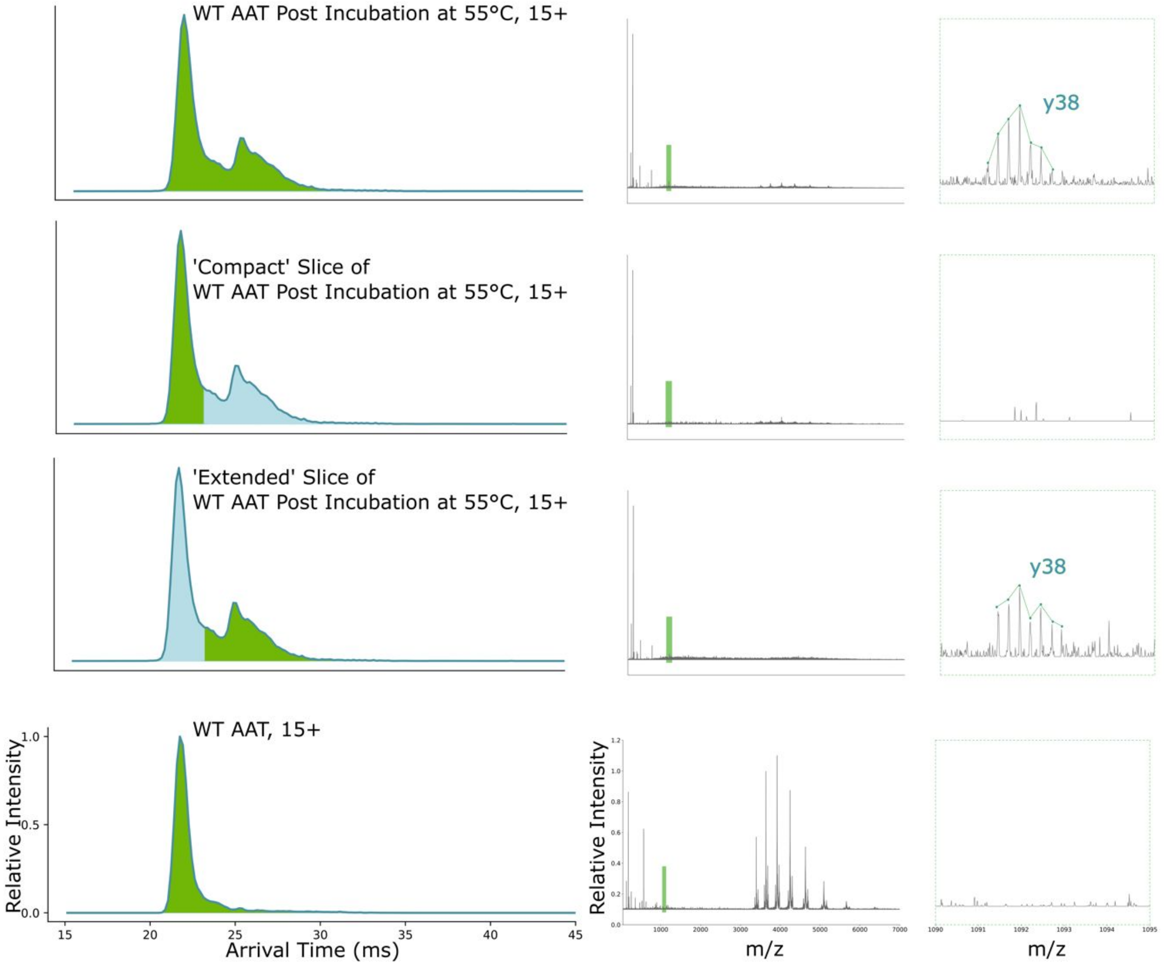

Supplementary Figure 3: Investigation of C-terminal release. The 15+ monomer of AAT_{WT} and AAT_{HEAT} were isolated and subjected to ECD. Although there are a low number of fragments for this charge state, the presence of the 'diagnostic' y38 ion indicates a released C-terminus in the heated sample that is not present in the WT sample. To further investigate this, the compact (native-like) and extended (intermediate-like) were conformationally selected before ECD. The compact conformer shows no y38 fragment, while the extended slice has the y38 fragment.

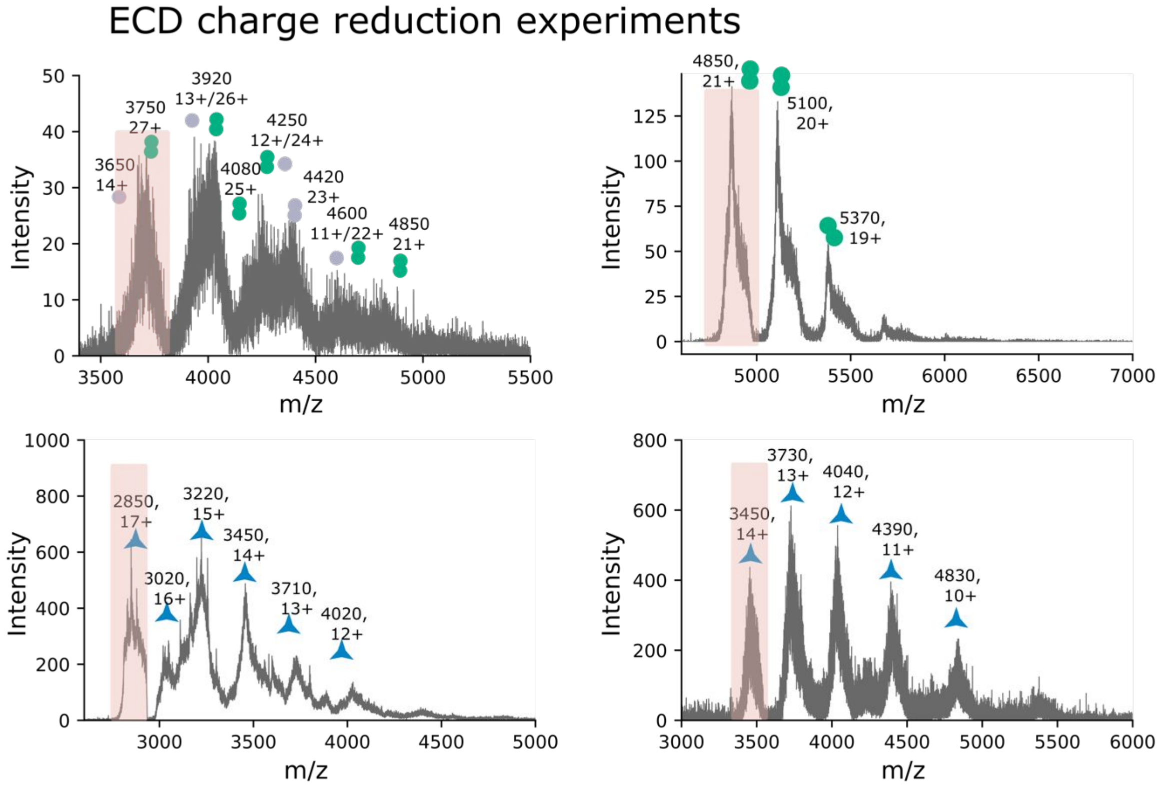

Supplementary Figure 4: Species were identified using the cIM-qToF following charge reduction in the ECD cell. The first peak in each graph is the quadrupole-isolated species while the following peaks have been charge-reduced, allowing for deconvolution of their precursor; values for *m/z* and charge are labelled, with each symbol corresponding to the species identified in the full mass spectrum and mass in Figure 5B.

Supplementary Tables

Supplementary Table 1: ECD fragment Assignment table for AAT_{HEAT}, Repeat 1. Data were acquired for the 2980 peak isolated in the quadrupole and subjected to ECD fragmentation with supplemental energy in the transfer of 30V to release fragments. Fragment peaks were deconvoluted and assigned using ExDViewer (Agilent)(ID:17Mon_ECD_12_10)[61].

Name	Residue Index	Amplitude	Molecular Composition	Mono Weight	Avg Weight	IonScore	Total Intensity	Avg PPM Error
**w10 2+**	383	2.45	C45H77N13O14	512.79	513.08	7.11	0.23	7.88
**w17 2+**	376	3.05	C83H137N21O23S	915.00	915.59	4.46	0.43	7.58
**w18 2+**	375	3.62	C87H143N23O25S	972.02	972.64	8.15	0.53	3.36
**w19 2+**	374	9.9	C92H151N25O27S	1036.05	1036.71	4.12	1.17	9.94
**w21 2+**	372	2.3	C103H169N27O31S	1157.12	1157.84	7.72	0.55	9.94
**w21 3+**	372	3.04	C103H169N27O31S	771.75	772.23	6.74	0.37	11.04
**w28 3+**	365	4.54	C148H235N35O38S2	1059.24	1059.94	7.60	0.80	8.58
**w32 3+**	361	2.3	C172H271N41O43S2	1222.00	1222.80	4.02	0.71	3.33
**w32 4+**	361	14.6	C172H271N41O43S2	916.75	917.35	12.33	2.47	8.11

**w37 4+**	356	2.75	C196H308N46O51S2	1047.57	1048.25	3.84	0.63	15.38
**w5 1+**	388	6.84	C23H38N6O8	527.28	527.57	4.55	0.38	8.26
**w7 1+**	386	3.23	C33H55N9O11	754.41	754.84	7.12	0.20	12.62
**y10 1+**	383	3.37	C47H84N14O14S	1101.61	1102.32	7.19	0.43	3.97
**y10 2+**	383	2.62	C47H84N14O14S	551.31	551.66	6.65	0.27	15.14
**y13 1+**	380	3	C67H111N17O17S	1458.81	1459.77	5.70	0.25	9.83
**y13 2+**	380	3.67	C67H111N17O17S	729.91	730.39	6.58	0.41	4.21
**y14 2+**	379	4.06	C70H116N18O19S	773.43	773.93	10.29	0.54	9.13
**y15 2+**	378	3.88	C76H128N20O20S	837.47	838.01	5.88	0.51	6.85
**y16 2+**	377	3.99	C80H135N21O22S	888.00	888.56	8.16	0.54	9.10
**y16-H2O 2+**	377	8.02	C80H133N21O21S	878.99	879.56	8.58	1.30	-4.98
**y17 2+**	376	4.28	C84H141N23O24S	945.02	945.61	9.37	0.62	4.62
**y18 2+**	375	5.28	C89H149N25O26S	1009.05	1009.68	7.66	0.81	9.69
**y18 3+**	375	3.01	C89H149N25O26S	673.03	673.45	4.20	0.32	11.37
**y19 2+**	374	4.45	C94H156N26O29S	1073.57	1074.24	6.84	0.81	11.34
**y20 2+**	373	3.46	C100H167N27O30S	1130.11	1130.82	6.19	0.75	4.67
**y21 2+**	372	2.54	C105H176N28O31S2	1195.63	1196.41	8.10	0.76	8.85
**y22 2+**	371	2.68	C111H187N29O32S2	1252.17	1252.99	6.84	0.68	11.66
**y26 2+**	367	4.03	C139H221N33O36S2	1497.30	1498.29	9.34	0.68	9.80
**y26 3+**	367	9.46	C139H221N33O36S2	998.54	999.20	9.27	1.84	12.48
**y27 3+**	366	3.37	C145H233N35O37S2	1041.24	1041.92	4.14	0.58	7.50
**y28 3+**	365	2.52	C149H239N37O39S2	1079.25	1079.95	3.49	0.66	3.79
**y29 3+**	364	3.46	C158H248N38O40S2	1128.27	1129.02	7.23	0.99	9.59
**y30 4+**	363	5.76	C164H260N40O41S2	878.48	879.06	6.98	1.20	9.33
**y31 3+**	362	2.45	C169H269N41O42S2	1203.99	1204.78	7.13	0.69	5.16
**y33 3+**	360	2.67	C179H283N43O46S2	1279.36	1280.19	8.57	0.95	9.12
**y33 4+**	360	6.29	C179H283N43O46S2	959.77	960.39	10.55	1.16	7.58
**y34 3+**	359	5.34	C184H290N44O47S2	1311.71	1312.56	9.92	1.66	9.51
**y34 4+**	359	15.75	C184H290N44O47S2	984.03	984.67	11.46	3.04	11.93
**y34 5+**	359	3.26	C184H290N44O47S2	787.43	787.94	7.30	0.65	8.75
**y36 4+**	357	4.89	C193H306N46O50S2	1034.06	1034.73	9.42	1.05	6.57
**y37 4+**	356	2.77	C198H315N47O51S3	1066.82	1067.53	3.55	0.77	0.41
**y38 3+**	355	4.49	C203H322N48O52S3	1454.45	1455.41	8.30	1.01	11.15
**y38 4+**	355	20.98	C203H322N48O52S3	1091.09	1091.81	12.92	5.43	10.42
**y38 5+**	355	17.25	C203H322N48O52S3	873.07	873.65	12.72	2.93	8.38
**y39 4+**	354	3.48	C209H333N49O53S3	1119.36	1120.10	7.82	1.05	10.88
**y4 1+**	389	10.9	C20H36N6O7	473.27	473.53	10.27	0.63	18.22
**y40 3+**	353	2.83	C212H338N50O54S3	1515.82	1516.82	7.98	0.73	6.59
**y40 4+**	353	5.42	C212H338N50O54S3	1137.12	1137.87	9.95	1.25	6.60
**y40 5+**	353	2.83	C212H338N50O54S3	909.90	910.50	3.26	0.59	6.22
**y5 1+**	388	6	C24H42N8O9	587.31	587.62	10.05	0.36	12.08

**y6 1+**	387	5.82	C29H51N9O10	686.38	686.77	9.83	0.41	10.56
**y7 1+**	386	9.22	C34H60N10O11	785.45	785.90	10.90	0.62	15.21
**y9 1+**	384	4.37	C42H75N13O13	970.57	971.12	6.63	0.37	14.59
**z10 1+**	383	4.14	C47H82N13O14S	1085.59	1086.30	8.44	0.67	6.47
**z10 2+**	383	3.03	C47H82N13O14S	543.30	543.65	7.81	0.36	6.07
**z11 1+**	382	5.14	C56H91N14O15S	1232.66	1233.47	9.25	1.09	8.42
**z11 2+**	382	5.09	C56H91N14O15S	616.83	617.24	10.46	0.55	11.83
**z12 1+**	381	5.76	C62H102N15O16S	1345.74	1346.62	9.28	1.00	10.58
**z12 2+**	381	4.28	C62H102N15O16S	673.37	673.81	8.12	0.40	7.96
**z14 1+**	379	6.04	C70H114N17O19S	1529.83	1530.83	9.47	0.92	8.54
**z14 2+**	379	10.76	C70H114N17O19S	765.42	765.92	13.97	1.21	8.59
**z15 2+**	378	11.19	C76H126N19O20S	829.46	830.00	11.73	1.43	7.84
**z16 2+**	377	6.9	C80H133N20O22S	879.99	880.55	9.33	0.93	9.15
**z17 2+**	376	12.87	C84H139N22O24S	937.01	937.60	11.44	1.49	8.95
**z17 3+**	376	9.74	C84H139N22O24S	625.01	625.40	11.19	1.10	7.45
**z18 2+**	375	13.28	C89H147N24O26S	1001.04	1001.67	10.10	1.75	9.94
**z18 3+**	375	9.61	C89H147N24O26S	667.70	668.11	12.57	1.07	5.65
**z19 2+**	374	6.81	C94H154N25O29S	1065.56	1066.23	8.42	1.20	10.07
**z19 3+**	374	2.92	C94H154N25O29S	710.71	711.16	6.36	0.49	8.82
**z2 1+**	391	6.44	C11H20N3O4	259.15	259.30	9.27	0.61	11.04
**z21 2+**	372	4.48	C105H174N27O31S2	1187.62	1188.40	7.53	1.22	11.80
**z21 3+**	372	4.23	C105H174N27O31S2	792.08	792.60	8.72	0.69	9.28
**z22 2+**	371	4.59	C111H185N28O32S2	1244.16	1244.98	8.19	1.22	7.60
**z22 3+**	371	3.73	C111H185N28O32S2	829.78	830.32	5.98	0.60	8.98
**z23 2+**	370	3.17	C120H194N29O33S2	1317.70	1318.57	6.88	0.92	12.63
**z24 2+**	369	3.83	C125H203N30O34S2	1367.23	1368.14	9.41	1.03	8.07
**z24 3+**	369	3.1	C125H203N30O34S2	911.82	912.43	5.43	0.55	10.69
**z25 2+**	368	9.23	C134H212N31O35S2	1440.77	1441.72	10.85	1.70	10.82
**z25 3+**	368	6.65	C134H212N31O35S2	960.85	961.48	8.96	1.19	6.64
**z27 2+**	366	9.01	C145H231N34O37S2	1553.34	1554.36	9.54	1.62	7.69
**z27 3+**	366	9.56	C145H231N34O37S2	1035.90	1036.58	8.54	2.06	9.74
**z28 2+**	365	5.98	C149H237N36O39S2	1610.36	1611.41	11.73	1.05	10.41
**z28 3+**	365	9.13	C149H237N36O39S2	1073.91	1074.61	10.43	1.73	9.10
**z28 4+**	365	2.61	C149H237N36O39S2	805.69	806.21	6.37	0.48	11.19
**z29 2+**	364	5.21	C158H246N37O40S2	1683.90	1685.01	6.83	0.95	8.55
**z29 3+**	364	5.1	C158H246N37O40S2	1122.93	1123.67	8.60	1.51	9.07
**z30 3+**	363	7.07	C164H258N39O41S2	1165.63	1166.40	9.22	2.14	9.14
**z30 4+**	363	4.29	C164H258N39O41S2	874.48	875.05	7.97	0.93	10.26
**z31 3+**	362	5.72	C169H267N40O42S2	1198.65	1199.44	10.51	1.63	9.89
**z31 4+**	362	3.36	C169H267N40O42S2	899.24	899.83	8.19	0.72	8.81
**z32 3+**	361	4.74	C174H274N41O45S2	1241.67	1242.48	10.92	1.39	11.22
**z32 4+**	361	3.76	C174H274N41O45S2	931.50	932.11	8.91	0.91	12.55

**z35 3+**	358	4.83	C190H299N44O48S2	1344.07	1344.94	10.74	1.51	9.06
**z35 4+**	358	3.65	C190H299N44O48S2	1008.30	1008.96	6.78	0.88	7.37
**z36 3+**	357	3.65	C193H304N45O50S2	1373.08	1373.96	9.36	1.29	8.60
**z36 4+**	357	5.21	C193H304N45O50S2	1030.06	1030.73	9.28	1.01	9.84
**z37 3+**	356	3.88	C198H313N46O51S3	1416.76	1417.70	9.07	1.02	7.55
**z37 4+**	356	3.03	C198H313N46O51S3	1062.82	1063.53	7.04	0.83	11.55
**z39 3+**	354	6.1	C209H331N48O53S3	1486.80	1487.79	10.20	1.42	10.97
**z39 4+**	354	5.05	C209H331N48O53S3	1115.35	1116.09	10.84	1.40	10.11
**z40 3+**	353	6.9	C212H336N49O54S3	1510.48	1511.48	10.23	1.61	11.19
**z40 4+**	353	6.32	C212H336N49O54S3	1133.11	1133.86	11.36	1.60	10.58
**z41 3+**	352	3.2	C217H343N50O57S3	1553.50	1554.52	4.02	0.93	8.60
**z41 4+**	352	3.42	C217H343N50O57S3	1165.37	1166.14	6.00	1.39	7.09
**z41 5+**	352	2.8	C217H343N50O57S3	932.50	933.11	5.06	0.86	9.61
**z42 4+**	351	2.57	C223H354N51O58S3	1193.64	1194.43	9.58	1.28	7.03
**z42 5+**	351	4.28	C223H354N51O58S3	955.12	955.75	8.27	0.92	10.94
**z43 4+**	350	3.29	C232H363N52O59S3	1230.41	1231.23	6.25	1.47	7.89
**z44 3+**	349	4.49	C237H372N53O60S4	1683.89	1685.03	7.41	1.08	9.34
**z44 4+**	349	6.46	C237H372N53O60S4	1263.17	1264.03	10.82	2.26	10.36
**z44 5+**	349	9.3	C237H372N53O60S4	1010.74	1011.42	10.84	2.19	11.76
**z45 4+**	348	4.15	C240H377N54O61S4	1280.93	1281.79	10.56	1.40	10.16
**z45 5+**	348	7.03	C240H377N54O61S4	1024.95	1025.64	8.82	1.69	8.43
**z46 4+**	347	2.23	C242H380N55O62S4	1295.19	1296.06	6.58	0.87	5.93
**z5 1+**	388	31.59	C24H40N7O9	571.30	571.60	11.79	2.62	9.39
**z6 1+**	387	5.6	C29H49N8O10	670.36	670.75	10.12	0.46	15.10
**z7 1+**	386	8.78	C34H58N9O11	769.43	769.88	11.79	0.73	8.68
**z8 1+**	385	9.12	C40H70N11O12	897.53	898.05	9.58	0.85	6.17
**z8 2+**	385	14.71	C40H70N11O12	449.27	449.53	11.92	1.01	6.91

Supplementary Table 2: ECD fragment Assignment table for AAT_{HEAT}, Repeat 2. Data were acquired for the 2980 peak isolated in the quadrupole and subjected to ECD fragmentation with supplemental energy in the transfer of 30V to release fragments. Fragment peaks were deconvoluted and assigned using ExDViewer (Agilent) (ID: HIP_17Mon_12_07)[61].

Name	Residue Index	Amplitude	Molecular Composition	Mono Weight	Avg Weight	IonScore	Total Intensity	Avg PPM Error
**c14 2+**	13	3.37	C57H92N18O27	731.33	731.72	5.99	0.27	5.68
**c15 2+**	14	4.54	C63H99N21O28	799.86	800.29	8.16	0.54	7.27
**c16 2+**	15	5.03	C69H106N24O29	868.39	868.87	6.96	0.60	6.24
**c23 3+**	22	4.61	C106H154N34O42	859.37	859.86	9.45	0.63	6.92
**c28 4+**	27	3.37	C131H197N41O49	783.11	783.56	5.34	0.51	7.83

**c32 4+**	31	8.22	C149H226N46O56	889.91	890.42	9.41	1.49	5.68
**c35 4+**	34	4.05	C170H249N49O59	981.21	981.78	7.51	0.93	1.09
**c39 5+**	38	4.46	C194H286N56O65	889.02	889.54	7.44	0.89	7.42
**c4 1+**	3	2.89	C19H30N6O9	487.21	487.47	6.53	0.19	17.42
**c5 1+**	4	5.13	C21H33N7O10	544.24	544.52	6.90	0.31	16.17
**c6 1+**	5	53.30	C25H38N8O13	659.26	659.62	14.59	2.90	-11.66
**w10 2+**	383	4.25	C45H77N13O14	512.79	513.08	10.79	0.32	6.70
**w12 2+**	381	3.66	C59H95N15O16S	651.85	652.27	8.48	0.36	6.31
**w17 2+**	376	4.24	C83H137N21O23S	915.00	915.59	4.87	0.62	-4.09
**w18 2+**	375	2.20	C87H143N23O25S	972.02	972.64	5.99	0.59	5.26
**w18 3+**	375	4.55	C87H143N23O25S	648.35	648.76	10.87	0.67	3.55
**w21 3+**	372	5.95	C103H169N27O31S	771.75	772.23	9.69	0.73	7.15
**w22 3+**	371	5.28	C108H178N28O32S2	815.43	815.96	9.02	0.61	4.15
**w32 4+**	361	17.32	C172H271N41O43S2	916.75	917.35	11.75	3.01	5.12
**w41 4+**	352	1.56	C215H340N50O55S3	1150.62	1151.38	3.35	0.85	9.14
**w44 5+**	349	3.72	C235H367N53O60S3	998.54	999.20	6.65	1.52	8.95
**y10 2+**	383	3.20	C47H84N14O14S	551.31	551.66	7.74	0.26	6.34
**y11 2+**	382	3.37	C56H93N15O15S	624.84	625.25	6.19	0.28	4.64
**y13 1+**	380	2.19	C67H111N17O17S	1458.81	1459.77	5.42	0.28	2.68
**y13 2+**	380	4.30	C67H111N17O17S	729.91	730.39	6.92	0.47	2.91
**y14 2+**	379	5.43	C70H116N18O19S	773.43	773.93	11.42	0.58	1.50
**y15 2+**	378	5.55	C76H128N20O20S	837.47	838.01	7.80	0.74	-0.17
**y16 2+**	377	3.86	C80H135N21O22S	888.00	888.56	5.84	0.54	13.49
**y17 2+**	376	4.09	C84H141N23O24S	945.02	945.61	7.03	0.79	-0.19
**y18 2+**	375	6.11	C89H149N25O26S	1009.05	1009.68	6.78	1.49	9.47
**y18 3+**	375	6.00	C89H149N25O26S	673.03	673.45	6.88	0.66	9.94
**y19 2+**	374	3.64	C94H156N26O29S	1073.57	1074.24	5.85	1.30	6.89
**y20 3+**	373	2.94	C100H167N27O30S	753.74	754.21	6.49	0.43	6.09
**y21 2+**	372	3.12	C105H176N28O31S2	1195.63	1196.41	5.67	1.28	9.35
**y21 3+**	372	3.70	C105H176N28O31S2	797.42	797.94	9.25	0.58	5.25
**y26 3+**	367	6.77	C139H221N33O36S2	998.54	999.20	8.89	2.30	6.96
**y27 3+**	366	2.46	C145H233N35O37S2	1041.24	1041.92	8.00	0.91	7.56
**y28 3+**	365	2.39	C149H239N37O39S2	1079.25	1079.95	7.45	0.95	2.14
**y29 3+**	364	2.36	C158H248N38O40S2	1128.27	1129.02	6.54	1.08	1.20
**y3 1+**	390	2.69	C15H29N5O6	376.22	376.42	10.03	0.13	4.64
**y30 3+**	363	2.61	C164H260N40O41S2	1170.97	1171.74	6.18	1.20	5.35
**y30 4+**	363	8.20	C164H260N40O41S2	878.48	879.06	7.01	1.61	5.93

**y31 3+**	362	2.07	C169H269N41O42S2	1203.99	1204.78	4.51	0.86	5.31
**y31 4+**	362	4.06	C169H269N41O42S2	903.25	903.84	9.12	0.84	3.54
**y33 4+**	360	5.86	C179H283N43O46S2	959.77	960.39	9.69	1.79	5.75
**y34 3+**	359	5.53	C184H290N44O47S2	1311.71	1312.56	11.12	2.24	6.24
**y34 4+**	359	18.03	C184H290N44O47S2	984.03	984.67	11.56	4.72	5.58
**y36 3+**	357	2.43	C193H306N46O50S2	1378.42	1379.31	7.73	1.01	4.36
**y36 4+**	357	3.70	C193H306N46O50S2	1034.06	1034.73	7.03	1.55	3.82
**y37 4+**	356	2.66	C198H315N47O51S3	1066.82	1067.53	3.91	1.30	6.94
**y38 4+**	355	21.67	C203H322N48O52S3	1091.09	1091.81	11.76	7.83	6.73
**y38 5+**	355	33.48	C203H322N48O52S3	873.07	873.65	12.89	6.34	4.92
**y38 6+**	355	5.80	C203H322N48O52S3	727.73	728.21	8.61	1.45	6.31
**y39 4+**	354	3.72	C209H333N49O53S3	1119.36	1120.10	8.53	1.72	5.07
**y39 5+**	354	4.14	C209H333N49O53S3	895.69	896.28	7.73	0.96	7.65
**y4 1+**	389	11.97	C20H36N6O7	473.27	473.53	11.59	0.76	17.27
**y40 3+**	353	5.29	C212H338N50O54S3	1515.82	1516.82	9.20	1.39	7.37
**y40 4+**	353	5.17	C212H338N50O54S3	1137.12	1137.87	10.50	2.02	5.22
**y40 5+**	353	5.64	C212H338N50O54S3	909.90	910.50	9.42	1.27	4.62
**y6 1+**	387	6.37	C29H51N9O10	686.38	686.77	9.86	0.45	7.25
**y8 2+**	385	2.89	C40H72N12O12	457.28	457.54	8.46	0.19	8.58
**z10 1+**	383	4.36	C47H82N13O14S	1085.59	1086.30	7.04	0.93	8.09
**z10 2+**	383	3.88	C47H82N13O14S	543.30	543.65	8.70	0.45	8.03
**z11 1+**	382	5.08	C56H91N14O15S	1232.66	1233.47	8.11	1.27	11.05
**z11 2+**	382	7.89	C56H91N14O15S	616.83	617.24	11.54	0.92	6.43
**z12 1+**	381	6.47	C62H102N15O16S	1345.74	1346.62	7.89	1.83	9.93
**z12 2+**	381	7.99	C62H102N15O16S	673.37	673.81	11.16	0.77	5.87
**z14 1+**	379	5.71	C70H114N17O19S	1529.83	1530.83	8.52	0.99	10.33
**z14 2+**	379	18.79	C70H114N17O19S	765.42	765.92	15.00	2.14	5.27
**z15 2+**	378	16.96	C76H126N19O20S	829.46	830.00	11.92	2.21	4.55
**z15 3+**	378	2.38	C76H126N19O20S	553.31	553.67	3.69	0.34	4.67
**z16 2+**	377	14.05	C80H133N20O22S	879.99	880.55	11.24	1.62	2.40
**z16 3+**	377	6.60	C80H133N20O22S	586.99	587.37	12.99	0.81	7.08
**z17 2+**	376	13.60	C84H139N22O24S	937.01	937.60	10.55	2.57	5.38
**z17 3+**	376	12.27	C84H139N22O24S	625.01	625.40	12.67	1.62	6.64
**z18 2+**	375	12.48	C89H147N24O26S	1001.04	1001.67	11.20	2.67	6.79
**z18 3+**	375	14.10	C89H147N24O26S	667.70	668.11	14.53	1.86	3.87
**z19 2+**	374	5.54	C94H154N25O29S	1065.56	1066.23	8.45	1.90	10.24
**z19 3+**	374	7.96	C94H154N25O29S	710.71	711.16	11.89	0.98	6.90
**z2 1+**	391	9.39	C11H20N3O4	259.15	259.30	9.52	0.50	16.67
**z20 3+**	373	4.22	C100H165N26O30S	748.40	748.87	10.16	0.66	7.33
**z21 2+**	372	4.81	C105H174N27O31S2	1187.62	1188.40	9.16	2.01	9.55
**z21 3+**	372	9.92	C105H174N27O31S2	792.08	792.60	11.64	1.31	6.12
**z22 2+**	371	5.46	C111H185N28O32S2	1244.16	1244.98	10.00	1.74	9.39
**z22 3+**	371	8.24	C111H185N28O32S2	829.78	830.32	6.96	1.13	6.31
**z23 2+**	370	2.54	C120H194N29O33S2	1317.70	1318.57	7.11	1.01	7.32

**z23 3+**	370	6.58	C120H194N29O33S2	878.80	879.38	5.76	1.01	11.55
**z24 2+**	369	4.71	C125H203N30O34S2	1367.23	1368.14	7.60	1.55	3.96
**z24 3+**	369	7.90	C125H203N30O34S2	911.82	912.43	10.30	1.31	5.33
**z25 2+**	368	9.85	C134H212N31O35S2	1440.77	1441.72	11.68	2.30	6.87
**z25 3+**	368	9.82	C134H212N31O35S2	960.85	961.48	11.26	2.44	6.82
**z27 2+**	366	9.60	C145H231N34O37S2	1553.34	1554.36	10.92	2.42	7.16
**z27 3+**	366	11.70	C145H231N34O37S2	1035.90	1036.58	9.56	3.69	6.46
**z27 4+**	366	3.42	C145H231N34O37S2	777.17	777.69	8.54	0.62	4.08
**z28 2+**	365	8.11	C149H237N36O39S2	1610.36	1611.41	10.54	1.56	6.57
**z28 3+**	365	11.66	C149H237N36O39S2	1073.91	1074.61	11.07	3.67	5.92
**z28 4+**	365	5.12	C149H237N36O39S2	805.69	806.21	11.64	0.83	6.77
**z29 2+**	364	8.01	C158H246N37O40S2	1683.90	1685.01	6.69	1.23	5.57
**z29 3+**	364	8.87	C158H246N37O40S2	1122.93	1123.67	9.30	3.43	5.76
**z29 4+**	364	5.28	C158H246N37O40S2	842.45	843.01	10.43	1.10	6.20
**z3 1+**	390	7.39	C15H27N4O6	360.20	360.40	7.03	0.54	10.54
**z30 3+**	363	7.28	C164H258N39O41S2	1165.63	1166.40	9.07	3.73	6.32
**z30 4+**	363	8.00	C164H258N39O41S2	874.48	875.05	11.24	1.60	5.95
**z31 3+**	362	6.73	C169H267N40O42S2	1198.65	1199.44	11.53	2.85	7.07
**z31 4+**	362	6.68	C169H267N40O42S2	899.24	899.83	8.43	1.31	4.61
**z32 3+**	361	5.06	C174H274N41O45S2	1241.67	1242.48	10.36	2.23	6.59
**z32 4+**	361	5.90	C174H274N41O45S2	931.50	932.11	9.47	1.62	6.89
**z35 3+**	358	6.10	C190H299N44O48S2	1344.07	1344.94	11.57	2.64	8.27
**z35 4+**	358	5.35	C190H299N44O48S2	1008.30	1008.96	9.35	2.21	1.80
**z36 3+**	357	5.47	C193H304N45O50S2	1373.08	1373.96	10.33	1.98	9.21
**z36 4+**	357	4.83	C193H304N45O50S2	1030.06	1030.73	9.20	1.98	6.01
**z37 3+**	356	5.23	C198H313N46O51S3	1416.76	1417.70	9.30	1.66	7.36
**z37 4+**	356	3.46	C198H313N46O51S3	1062.82	1063.53	9.08	2.03	6.40
**z39 3+**	354	6.12	C209H331N48O53S3	1486.80	1487.79	9.49	2.14	8.41
**z39 4+**	354	6.04	C209H331N48O53S3	1115.35	1116.09	11.02	2.85	7.08
**z39 5+**	354	3.84	C209H331N48O53S3	892.48	893.08	7.42	0.93	5.40
**z40 3+**	353	9.13	C212H336N49O54S3	1510.48	1511.48	10.30	2.38	6.76
**z40 4+**	353	9.02	C212H336N49O54S3	1133.11	1133.86	11.07	3.76	7.34
**z40 5+**	353	7.12	C212H336N49O54S3	906.69	907.29	11.28	1.41	2.92
**z41 3+**	352	2.58	C217H343N50O57S3	1553.50	1554.52	8.55	0.95	10.91
**z41 4+**	352	3.40	C217H343N50O57S3	1165.37	1166.14	7.67	2.13	8.01
**z41 5+**	352	5.37	C217H343N50O57S3	932.50	933.11	9.50	1.53	8.71
**z42 3+**	351	2.95	C223H354N51O58S3	1591.19	1592.24	7.50	0.74	5.23
**z42 4+**	351	3.11	C223H354N51O58S3	1193.64	1194.43	9.27	1.96	7.31
**z42 5+**	351	5.51	C223H354N51O58S3	955.12	955.75	10.01	1.87	6.08
**z43 4+**	350	4.09	C232H363N52O59S3	1230.41	1231.23	7.55	1.97	7.01
**z43 5+**	350	5.80	C232H363N52O59S3	984.53	985.18	7.96	2.25	4.77
**z44 3+**	349	6.33	C237H372N53O60S4	1683.89	1685.03	6.99	1.36	6.07
**z44 4+**	349	8.16	C237H372N53O60S4	1263.17	1264.03	12.19	3.93	6.36
**z44 5+**	349	9.73	C237H372N53O60S4	1010.74	1011.42	10.96	4.15	6.86

**z45 4+**	348	4.36	C240H377N54O61S4	1280.93	1281.79	10.02	2.26	8.88
**z45 5+**	348	9.38	C240H377N54O61S4	1024.95	1025.64	11.64	3.97	8.10
**z5 1+**	388	39.29	C24H40N7O9	571.30	571.60	15.06	3.52	7.96
**z50 5+**	343	4.69	C257H404N59O69S4	1110.78	1111.52	7.72	2.82	4.48
**z6 1+**	387	7.51	C29H49N8O10	670.36	670.75	11.10	0.54	7.18
**z63 5+**	330	2.86	C318H507N76O87S4	1383.14	1384.04	7.71	1.67	7.56
**z63 6+**	330	3.96	C318H507N76O87S4	1152.78	1153.53	11.06	2.56	6.28
**z66 6+**	327	3.36	C333H535N80O91S4	1207.48	1208.27	7.91	1.88	2.32
**z7 1+**	386	13.90	C34H58N9O11	769.43	769.88	14.66	1.02	7.53
**z71 6+**	322	2.05	C358H577N86O98S4	1297.20	1298.04	7.90	1.29	4.31
**z71 8+**	322	1.93	C358H577N86O98S4	973.15	973.78	7.27	0.66	2.89
**z8 1+**	385	13.32	C40H70N11O12	897.53	898.05	9.09	1.18	8.81
**z8 2+**	385	18.62	C40H70N11O12	449.27	449.53	13.75	1.42	9.13
**z9 1+**	384	8.39	C42H73N12O13	954.55	955.10	7.11	0.90	9.29
**z9 2+**	384	3.72	C42H73N12O13	477.78	478.05	8.89	0.35	8.40

Supplementary Table 3: ECD fragment Assignment table for AAT_{HEAT}, Repeat 3. Data were acquired for the 2980 peak isolated in the quadrupole and subjected to ECD fragmentation with supplemental energy in the transfer of 30V to release fragments. Fragment peaks were deconvoluted and assigned using ExDViewer (Agilent)[61].

Name	Residue Index	Molecular Composition	Mono Weight	Avg Weight	IonScore	Total Intensity	Avg PPM Error
**c3 2+**	2	C14H22N4O7	180.08	180.18	5.74	5.33	5.11
**c4 2+**	3	C19H30N6O9	244.11	244.24	5.40	1.74	12.21
**c4-2(NH3) 2+**	3	C19H24N4O9	227.08	227.21	8.08	4.12	19.77
**c6 1+**	5	C25H38N8O13	659.26	659.62	15.11	1.91	-12.23
**c8 3+**	7	C31H48N10O15	267.78	267.93	5.12	1.65	-17.63
**w14 1+**	379	C70H113N17O18S	1512.82	1513.82	3.38	0.35	1.31
**w17 2+**	376	C83H137N21O23S	915.00	915.59	6.26	0.48	3.16
**w18 2+**	375	C87H143N23O25S	972.02	972.64	7.10	0.43	8.61
**w18 3+**	375	C87H143N23O25S	648.35	648.76	3.08	0.31	2.36
**w20 2+**	373	C98H160N26O30S	1107.58	1108.28	3.43	0.47	10.13
**w22 2+**	371	C108H178N28O32S2	1222.64	1223.44	6.09	0.59	5.69
**w28 3+**	365	C148H235N35O38S2	1059.24	1059.94	7.55	0.64	8.83
**w32 4+**	361	C172H271N41O43S2	916.75	917.35	11.54	1.35	6.31

**w5 1+**	388	C23H38N6O8	527.28	527.57	5.73	0.43	11.11
**y13 1+**	380	C67H111N17O17S	1458.81	1459.77	5.77	0.31	-1.10
**y13 2+**	380	C67H111N17O17S	729.91	730.39	7.05	0.42	0.90
**y14 2+**	379	C70H116N18O19S	773.43	773.93	9.44	0.37	3.60
**y15 2+**	378	C76H128N20O20S	837.47	838.01	6.56	0.46	0.28
**y16 2+**	377	C80H135N21O22S	888.00	888.56	6.14	0.42	10.14
**y17 2+**	376	C84H141N23O24S	945.02	945.61	8.60	0.55	4.02
**y18 2+**	375	C89H149N25O26S	1009.05	1009.68	8.97	0.79	6.80
**y18 3+**	375	C89H149N25O26S	673.03	673.45	6.87	0.40	5.16
**y19 2+**	374	C94H156N26O29S	1073.57	1074.24	7.25	0.80	8.43
**y20 2+**	373	C100H167N27O30S	1130.11	1130.82	8.51	0.75	4.50
**y21 2+**	372	C105H176N28O31S2	1195.63	1196.41	7.14	0.66	4.95
**y26 2+**	367	C139H221N33O36S2	1497.30	1498.29	5.28	0.59	-0.83
**y26 3+**	367	C139H221N33O36S2	998.54	999.20	9.35	1.21	7.28
**y27 3+**	366	C145H233N35O37S2	1041.24	1041.92	6.75	0.62	7.59
**y29 3+**	364	C158H248N38O40S2	1128.27	1129.02	7.38	0.78	5.77
**y30 3+**	363	C164H260N40O41S2	1170.97	1171.74	7.91	0.64	9.76
**y30 4+**	363	C164H260N40O41S2	878.48	879.06	7.01	0.62	5.99
**y31 3+**	362	C169H269N41O42S2	1203.99	1204.78	3.18	0.68	3.52
**y33 4+**	360	C179H283N43O46S2	959.77	960.39	9.14	1.15	2.07
**y34 3+**	359	C184H290N44O47S2	1311.71	1312.56	10.24	1.51	9.59
**y34 4+**	359	C184H290N44O47S2	984.03	984.67	10.89	1.88	7.00
**y36 4+**	357	C193H306N46O50S2	1034.06	1034.73	9.20	0.69	5.60
**y38 3+**	355	C203H322N48O52S3	1454.45	1455.41	7.15	0.85	6.48
**y38 4+**	355	C203H322N48O52S3	1091.09	1091.81	10.56	2.50	7.50
**y38 5+**	355	C203H322N48O52S3	873.07	873.65	10.92	1.58	6.52
**y39 4+**	354	C209H333N49O53S3	1119.36	1120.10	7.61	0.77	1.99
**y40 3+**	353	C212H338N50O54S3	1515.82	1516.82	8.73	0.90	-1.33
**y40 4+**	353	C212H338N50O54S3	1137.12	1137.87	9.50	1.07	4.68
**y5 1+**	388	C24H42N8O9	587.31	587.62	7.83	0.34	13.44
**y7 1+**	386	C34H60N10O11	785.45	785.90	11.75	0.60	6.39
**y9 1+**	384	C42H75N13O13	970.57	971.12	6.15	0.32	15.83
**z10 1+**	383	C47H82N13O14S	1085.59	1086.30	7.89	0.71	2.03
**z11 1+**	382	C56H91N14O15S	1232.66	1233.47	9.26	1.12	4.80
**z11 2+**	382	C56H91N14O15S	616.83	617.24	9.57	0.52	3.61
**z12 1+**	381	C62H102N15O16S	1345.74	1346.62	9.18	0.91	6.74
**z12 2+**	381	C62H102N15O16S	673.37	673.81	9.86	0.39	2.53
**z14 1+**	379	C70H114N17O19S	1529.83	1530.83	8.73	0.98	5.18
**z14 2+**	379	C70H114N17O19S	765.42	765.92	14.07	1.21	6.12
**z15 2+**	378	C76H126N19O20S	829.46	830.00	10.83	1.21	7.33
**z16 2+**	377	C80H133N20O22S	879.99	880.55	11.28	1.00	4.83
**z16 3+**	377	C80H133N20O22S	586.99	587.37	3.96	0.42	-3.33
**z17 2+**	376	C84H139N22O24S	937.01	937.60	11.38	1.53	5.36
**z17 3+**	376	C84H139N22O24S	625.01	625.40	10.31	0.81	6.63

**z18 2+**	375	C89H147N24O26S	1001.04	1001.67	10.57	1.80	6.09
**z18 3+**	375	C89H147N24O26S	667.70	668.11	12.31	0.98	5.12
**z19 2+**	374	C94H154N25O29S	1065.56	1066.23	9.30	1.03	9.31
**z19 3+**	374	C94H154N25O29S	710.71	711.16	8.98	0.55	8.67
**z21 2+**	372	C105H174N27O31S2	1187.62	1188.40	10.20	1.12	9.55
**z21 3+**	372	C105H174N27O31S2	792.08	792.60	10.35	0.57	6.64
**z22 2+**	371	C111H185N28O32S2	1244.16	1244.98	10.18	1.08	4.61
**z22 3+**	371	C111H185N28O32S2	829.78	830.32	5.66	0.59	7.18
**z23 2+**	370	C120H194N29O33S2	1317.70	1318.57	9.17	0.77	8.49
**z24 2+**	369	C125H203N30O34S2	1367.23	1368.14	8.26	1.02	4.00
**z25 2+**	368	C134H212N31O35S2	1440.77	1441.72	10.00	1.64	5.13
**z25 3+**	368	C134H212N31O35S2	960.85	961.48	7.03	0.96	3.12
**z27 2+**	366	C145H231N34O37S2	1553.34	1554.36	11.11	1.85	5.01
**z27 3+**	366	C145H231N34O37S2	1035.90	1036.58	7.73	1.93	5.17
**z28 2+**	365	C149H237N36O39S2	1610.36	1611.41	11.08	1.13	2.46
**z28 3+**	365	C149H237N36O39S2	1073.91	1074.61	12.20	1.82	4.89
**z29 2+**	364	C158H246N37O40S2	1683.90	1685.01	4.88	0.97	7.12
**z29 3+**	364	C158H246N37O40S2	1122.93	1123.67	9.65	1.73	7.49
**z30 3+**	363	C164H258N39O41S2	1165.63	1166.40	10.00	2.25	5.70
**z30 4+**	363	C164H258N39O41S2	874.48	875.05	7.61	0.86	8.63
**z31 3+**	362	C169H267N40O42S2	1198.65	1199.44	11.32	1.59	4.65
**z31 4+**	362	C169H267N40O42S2	899.24	899.83	4.23	0.68	8.76
**z32 3+**	361	C174H274N41O45S2	1241.67	1242.48	11.07	1.54	7.36
**z32 4+**	361	C174H274N41O45S2	931.50	932.11	7.98	0.66	4.49
**z35 3+**	358	C190H299N44O48S2	1344.07	1344.94	11.93	1.68	8.59
**z35 4+**	358	C190H299N44O48S2	1008.30	1008.96	4.19	0.82	1.97
**z36 3+**	357	C193H304N45O50S2	1373.08	1373.96	9.80	1.35	8.57
**z37 3+**	356	C198H313N46O51S3	1416.76	1417.70	10.41	1.09	7.31
**z37 4+**	356	C198H313N46O51S3	1062.82	1063.53	6.33	0.58	3.36
**z39 3+**	354	C209H331N48O53S3	1486.80	1487.79	9.80	1.35	6.47
**z39 4+**	354	C209H331N48O53S3	1115.35	1116.09	9.20	1.10	6.32
**z40 3+**	353	C212H336N49O54S3	1510.48	1511.48	11.28	1.55	3.60
**z40 4+**	353	C212H336N49O54S3	1133.11	1133.86	10.75	1.64	5.67
**z40 5+**	353	C212H336N49O54S3	906.69	907.29	3.14	0.68	3.38
**z41 4+**	352	C217H343N50O57S3	1165.37	1166.14	6.93	1.02	8.75
**z42 4+**	351	C223H354N51O58S3	1193.64	1194.43	4.86	0.87	5.50
**z43 4+**	350	C232H363N52O59S3	1230.41	1231.23	10.13	1.24	6.01
**z43 5+**	350	C232H363N52O59S3	984.53	985.18	7.39	0.86	6.96
**z44 3+**	349	C237H372N53O60S4	1683.89	1685.03	7.41	1.25	7.50
**z44 4+**	349	C237H372N53O60S4	1263.17	1264.03	11.74	2.40	6.03
**z44 5+**	349	C237H372N53O60S4	1010.74	1011.42	9.75	1.54	4.08
**z45 4+**	348	C240H377N54O61S4	1280.93	1281.79	9.30	1.32	7.52
**z45 5+**	348	C240H377N54O61S4	1024.95	1025.64	9.19	1.30	5.04
**z46 4+**	347	C242H380N55O62S4	1295.19	1296.06	6.79	0.97	6.45

**z46 5+**	347	C242H380N55O62S4	1036.35	1037.05	6.71	1.01	3.45
**z5 1+**	388	C24H40N7O9	571.30	571.60	15.43	2.68	6.10
**z5-2(NH3) 2+**	388	C24H34N5O9	269.13	269.27	5.75	1.27	-12.54
**z6 1+**	387	C29H49N8O10	670.36	670.75	8.55	0.51	14.61
**z7 1+**	386	C34H58N9O11	769.43	769.88	11.91	0.73	2.51
**z8 1+**	385	C40H70N11O12	897.53	898.05	9.70	0.93	13.33
**z9 1+**	384	C42H73N12O13	954.55	955.10	8.54	0.79	7.58
**z9 2+**	384	C42H73N12O13	477.78	478.05	6.20	0.26	10.83

Supplementary Table 4: ECD Ion Matches for AAT isolated from ex-vivo liver polymers. Data were acquired for the 3450 peak (assigned to a mass of 48400 Da) isolated in the quadrupole and subjected to ECD fragmentation with supplemental energy in the transfer of 30V to release fragments. Fragment peaks were deconvoluted using ExDViewer (Agilent)[61] and assigned manually by comparing to predicted fragments of the AAT sequence produced using MS product[62].

Name	Ion Score	Total Intensity	PPM	Monoisotopic	Assigment	Residue	Residue Number
**#246.16 1+**	2.54	170.81	-0.40	246.16	b2	D	2
**#359.21 1+**	4.23	207.43	0.41	359.21	c3	P	3
**#443.35 1+**	6.65	389.72	-0.88	443.35	a4	Q	4
**#487.38 1+**	5.85	265.11	7.28	487.38	c4	Q	4
**#452.42 1+**	4.53	188.62	-0.59	452.42	b4-H20	Q	4
**#471.28 1+**	2.73	230.16	12.69	471.28	b4	Q	4
**#527.31 1+**	4.49	223.92	-1.45	527.31	b5	G	5
**#642.41 1+**	6.56	266.25	3.00	642.41	b6	D	6
**c7 2+**	4.81	196.58	-6.04	365.65	c7	A	7
**#1374.14 3+**	5.39	702.84	0.49	1373.47	c37	L	37
**#1511.22 3+**	9.51	1135.59	-0.80	1510.55	z40	A	355
**#1516.55 3+**	7.11	564.89	3.50	1515.88	y40	A	355
**#1487.52 3+**	5.53	712.26	4.50	1486.85	z39	I	356
**#1091.64 4+**	6.06	454.16	-3.74	1091.14	y38	P	357

**#1455.18 3+**	3.29	246.33	2.02	1454.51	y38	P	357
**#873.50 5+**	2.71	396.47	6.11	873.10	y38	P	357
**#1417.49 3+**	8.96	725.80	0.13	1416.82	z37	M	358
**#1344.80 3+**	8.90	1056.98	0.67	1344.13	z35	I	360
**#1008.34 4+**	1.84	291.22	3.31	1007.84	z35	I	360
**#984.58 4+**	2.21	289.55	2.12	984.07	y34	P	361
**#1312.12 3+**	1.75	457.64	-5.68	1311.45	y34	P	361
**#1311.77 3+**	1.71	474.02	-0.64	1311.10	y34	P	361
**#1242.39 3+**	4.79	719.58	-3.70	1241.72	z32	E	363
**#932.04 4+**	2.93	235.40	2.80	931.54	z32	E	363
**#1199.38 3+**	5.63	1061.75	-4.10	1198.71	z31	V	364
**#1166.35 3+**	9.27	1404.30	3.26	1165.68	z30	K	365
**#1749.02 2+**	3.05	278.53	-0.22	1748.02	z30	K	365
**#1123.32 3+**	9.36	741.54	-3.50	1122.99	z29	F	366
**#1684.48 2+**	7.76	725.77	0.17	1683.98	z29	F	366
**#1611.44 2+**	11.53	1227.44	0.88	1610.94	z28	N	367
**#1074.30 3+**	7.42	941.97	-3.96	1073.97	z28	N	367
**#1553.90 2+**	10.03	1570.10	2.59	1553.40	z27	K	368
**#1036.28 3+**	6.67	646.03	-5.53	1035.95	z27	K	368
**#1441.33 2+**	12.03	1975.21	2.03	1440.83	z25	F	370
**#961.22 3+**	9.10	685.93	1.59	960.89	z25	F	370
**#1367.79 2+**	5.52	437.25	3.93	1367.29	z24	V	371
**#1318.26 2+**	7.30	397.05	0.29	1317.75	z23	F	372
**#1244.72 2+**	9.55	865.29	1.02	1244.22	z22	L	373
**#1188.18 2+**	7.76	794.93	0.30	1187.68	z21	M	374

**#748.77 3+**	2.85	100.55	0.33	748.44	z20	I	375
**#1122.66 2+**	2.02	341.97	-4.32	1122.16	z20	I	375
**#1066.12 2+**	8.43	634.78	-5.63	1065.61	z19	E	376
**#711.07 3+**	3.53	146.61	-0.20	710.74	z19	E	376
**#1001.59 2+**	13.63	1377.22	-4.73	1001.09	z18	Q	377
**#668.06 3+**	8.60	332.79	4.75	667.72	z18	Q	377
**#937.56 2+**	10.81	1361.17	-4.53	937.06	z17	N	378
**#625.37 3+**	3.03	125.35	-6.45	625.04	z17	N	378
**#880.03 2+**	9.83	584.46	-2.33	880.03	z16	T	379
**#829.50 2+**	7.30	610.10	-4.19	829.50	z15	K	380
**#1658.00 1+**	5.01	300.53	1.15	1658.00	z15	K	380
**#837.51 2+**	2.43	175.54	-4.16	837.51	y15	K	380
**#765.45 2+**	11.12	799.50	1.98	765.45	z14	S	381
**#1529.90 1+**	9.44	1105.17	-4.90	1529.90	z14	S	381
**#729.94 2+**	2.82	101.14	-4.70	729.94	y13	P	382
**#1345.81 1+**	10.13	1516.84	2.17	1345.81	z12	L	383
**#673.40 2+**	5.54	241.41	5.52	673.40	z12	L	383
**#1232.71 1+**	11.10	952.35	-2.81	1232.71	z11	F	384
**#617.36 2+**	8.59	275.16	3.30	617.36	z11	F	384
**#1085.64 1+**	10.56	630.35	-1.19	1085.64	z10	M	385
**#1101.66 1+**	2.20	149.96	1.37	1101.66	y10	M	385
**#543.33 2+**	2.17	103.33	-5.15	543.33	z10	M	385
**#955.60 1+**	11.34	813.38	4.45	955.60	z9	G	386
**#970.61 1+**	2.13	114.84	0.50	970.61	y9	G	386
**#897.57 1+**	12.05	1122.34	3.59	897.57	z8	K	387

**#915.57 1+**	7.24	384.76	5.23	915.57	y8?	K	387
**#457.37 1+**	3.71	176.65	2.23	457.37	y8	K	387
**#768.49 1+**	5.45	831.03	5.57	768.49	y7-NH3	V	388
**#769.46 1+**	4.70	668.21	8.50	769.46	z7	V	388
**#785.48 1+**	3.94	152.66	1.19	785.48	y7	V	388
**#670.40 1+**	5.71	226.20	-6.27	670.40	z6	V	389
**#686.41 1+**	2.04	104.29	-3.90	686.41	y6	V	389
**#571.32 1+**	10.07	3054.47	-1.39	571.32	z5	N	390
**#587.34 1+**	2.40	93.49	-3.89	587.34	y5	N	390
**#473.29 1+**	3.17	341.44	5.37	473.29	y4	P	391
**#361.23 1+**	9.11	397.30	5.37	361.23	z3	T	392
**#260.17 1+**	5.05	878.17	8.70	260.17	z2	Q	393
**#258.29 1+**	4.08	590.82	1.21	258.29	y2-NH3	Q	393
**#275.18 1+**	3.41	187.95	-0.06	275.18	y2	Q	393
